# ESM2-Guided Context-Aware Annotation Completion Supplements Carbohydrate Metabolism Coverage in Silage Microbial Metagenomes

**DOI:** 10.3390/microorganisms14081848

**Published:** 2026-08-20

**Authors:** Jiewei Zhang, Xinyu Du, Jinbiao Tang, Xiaoning Dong, Xusheng Guo, Mingxue Li, Dongmei Xu

**Affiliations:** 1 School of Electrical Engineering, Northwest Minzu University, Lanzhou 730030, China; zhangjiewei@stu.xbmu.edu.cn (J.Z.); y241630432@stu.xbmu.edu.cn (X.D.); utopia_land@163.com (J.T.); dongxiaoning@stu.xbmu.edu.cn (X.D.); 2 School of Life Sciences, Lanzhou University, Lanzhou 730000, China; guoxsh07@lzu.edu.cn; 3 Probiotics and Life Health Institute, Lanzhou University, Lanzhou 730000, China

**Keywords:** silage microbiome, metagenomics, functional annotation, protein language model, genomic context, carbohydrate-active enzymes

## Abstract

Functional annotation gaps limit the interpretation of carbohydrate metabolism in silage microbiomes. We developed Context-Aware Annotation Completion (CAAC), a framework integrating ESM2 embeddings, genomic-neighborhood features, three-class classification, confidence-tiered neighbor voting, and Enzyme Commission (EC)-to-KEGG Orthology (KO) mapping. CAAC was applied to 21 metagenomes from uninoculated and *Lacticaseibacillus paracasei*-inoculated silages sampled before ensiling and at 7 and 90 days. Five-fold cross-validation yielded an F1-macro of 84.64% for negative, positive, and hard-sequence classification. Among 800,000 selected annotation-poor sequences, 545,671 Tier 1 or Tier 2 predictions passed the annotation-validity and EC-to-KO mapping criteria, of which 524,814 were eligible for sample-level annotation supplementation. After silage-focused filtering and KO–EC summarization, these predictions yielded 102 KO–EC features repeatedly detected across the silage metagenomes and increased coverage in 25 of 47 carbohydrate-metabolism pathways, mainly by recovering enzyme-level components related to starch and sucrose, cellulose and cellobiose, xylan and hemicellulose, and pectin and glucuronate metabolism. Taxon-linked analyses further revealed treatment- and stage-associated patterns in the taxonomic sources of the supplemented annotations. A database-derived temporal benchmark using the July 2025 CAZy release showed 94.94% Tier 1 family-level annotation-transfer consistency. CAAC extends the enzyme-level interpretation of under-annotated silage metagenomes, while the inferred assignments remain computational predictions requiring experimental validation.

## 1. Introduction

Silage production is a critical method for preserving forage and feed materials, with the quality and nutritional value of the preserved feed strongly influenced by microbial metabolic activity during fermentation [1]. Carbohydrates represent the primary substrates in silage raw materials, and their degradation and conversion substantially influence the efficiency of lactic acid fermentation, dry matter loss, and the ultimate palatability of the feed [2]. A comprehensive understanding of these intricate metabolic processes requires an in-depth analysis of community functional composition. However, traditional cultivation techniques are inherently limited in their ability to characterize the vast diversity of uncultured microorganisms present in the environment [3]. Metagenomic sequencing provides a culture-independent means to profile community-wide gene functions [4]. However, traditional functional annotation relies on homology-based sequence searches against databases such as CAZy (Carbohydrate-Active Enzymes) [5], KEGG (Kyoto Encyclopedia of Genes and Genomes) [6], and InterProScan [7], which routinely fail to assign functions to a substantial fraction of protein-coding genes [8]. In silage microbial metagenomes, annotation-poor sequences can constitute a substantial fraction of predicted genes, leaving critical gaps in the characterization of community carbohydrate metabolic potential.

Pretrained protein language models, notably ESM2 (Evolutionary Scale Modeling 2), offer an alternative by extracting high-dimensional features that capture structural and evolutionary information from raw amino acid sequences [9,10]. Recent studies have demonstrated their utility in downstream functional prediction tasks [10]. However, sequence embeddings alone may not reliably distinguish functionally ambiguous proteins, particularly for enzymes with weak homology to characterized families. Moreover, integrating computational predictions into existing annotation frameworks, so that they supplement rather than displace traditional methods, remains unresolved.

Genomic context provides complementary functional evidence. Functionally coupled genes tend to co-localize in prokaryotic genomes, a principle established by comparative genomics [11,12,13]. Yet, in metagenomic studies, assembly fragmentation severely limits neighborhood recovery [14], and existing methods rarely combine protein sequence features with genomic context in a systematic manner for poorly annotated sequences. These limitations are especially acute in silage microbiomes [1,3]. The taxonomic complexity of fermentation communities, combined with limited genomic characterization of many prevalent lineages, generates large pools of unannotated genes that may include key carbohydrate-active enzymes [1,3]. Their omission from annotation databases leads to incomplete descriptions of substrate utilization and product formation during ensiling.

To address these gaps, we developed Context-Aware Annotation Completion (CAAC), a computational framework that integrates ESM2 protein embeddings, genomic-context features, confidence-tiered prediction, and Enzyme Commission (EC)-to-KEGG Orthology (KO) mapping to supplement conventional KEGG annotations for poorly annotated silage metagenomic sequences. CAAC was designed to recover putative carbohydrate-related functions missed by homology-based annotation while preserving clear confidence tiers and integration criteria. These functional assignments represent computational predictions whose biological accuracy requires experimental validation.

## 2. Materials and Methods

### 2.1. Framework

**Figure 1:**
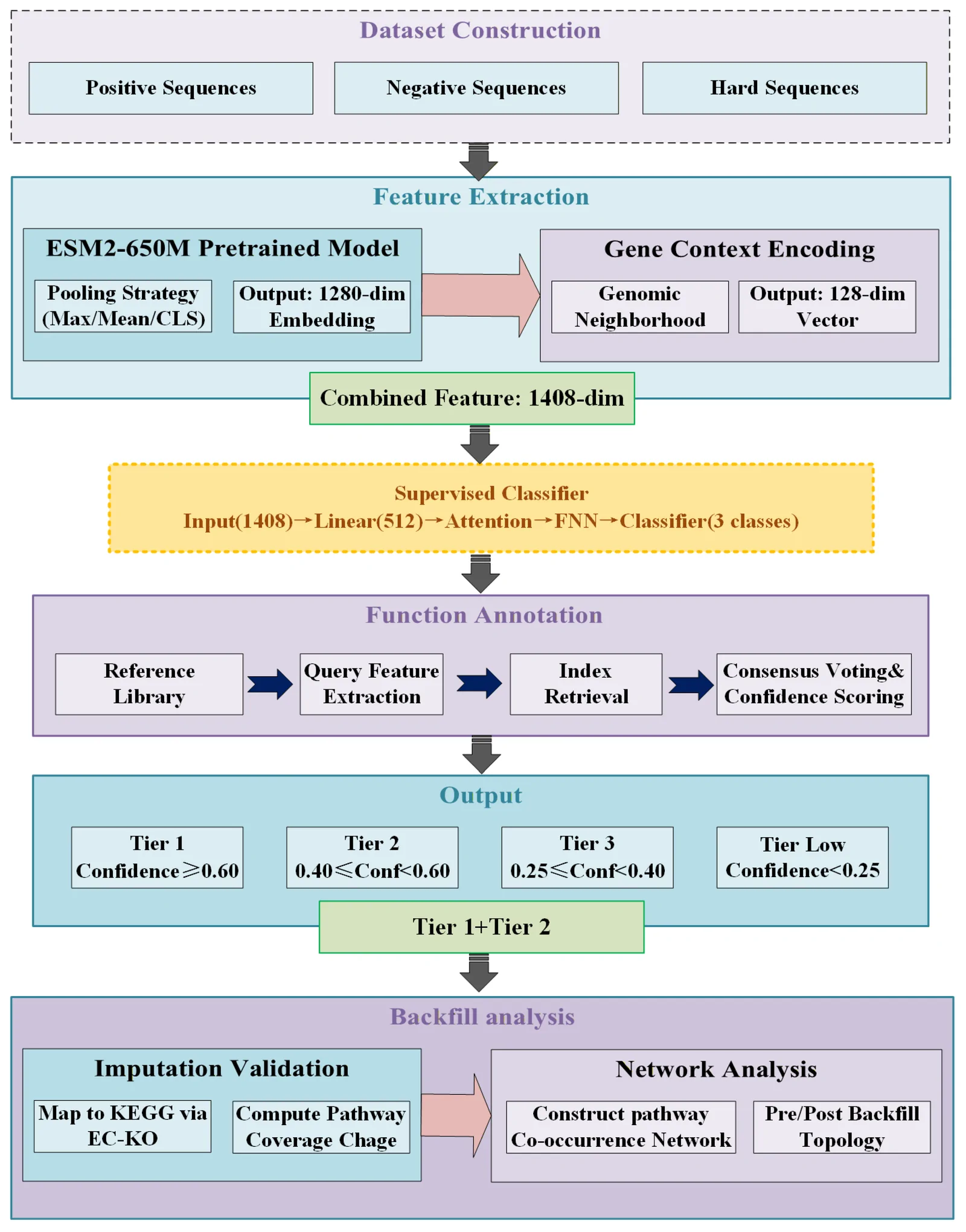
Overall workflow of the CAAC framework.

The CAAC framework was organized into three stages for predicting functions of poorly annotated proteins in silage microbial metagenomes [1,3] (Figure 1). First, positive, operational negative-control, and hard sequences were assembled, and hard sequences were partitioned into representatives for classifier training and members for downstream inference. Second, ESM2-derived protein embeddings [10] were fused with genomic-neighborhood features [11,12] to train a supervised three-class classifier. Third, sequences predicted as negative were excluded from catalytic EC/KO-oriented annotation transfer, whereas positive- and hard-class predictions underwent neighbor voting and confidence scoring. Tier 1 and Tier 2 assignments meeting the annotation-validity and EC-to-KO mapping criteria were integrated with the original KEGG annotations for pathway coverage and annotation-derived co-occurrence analyses.

### 2.2. Data Construction

The training set comprised positive, negative, and hard sequences (Figure 1). Positive sequences were obtained from the CAZy database [5] and from experimentally supported UniProt entries [15] with Protein Existence levels of 1 or 2. This class included sequences from glycoside hydrolase (GH), glycosyltransferase (GT), and carbohydrate esterase (CE) families, as well as enzymes with explicit EC numbers or carbohydrate metabolism-related Gene Ontology annotations [16]. After CD-HIT 4.8.1 clustering [17] at 90% sequence identity and 90% alignment coverage, 335,970 nonredundant positive representatives were retained.

The negative class was defined as an operational non-target control class for the catalytic EC/KO-oriented annotation-transfer task. It comprised housekeeping proteins, including ribosomal proteins, RNA polymerases, and DNA repair proteins, together with non-catalytic carbohydrate-binding module (CBM) sequences from CAZy. CBMs were included as non-catalytic controls because they are carbohydrate-associated binding modules but do not generally provide directly transferable catalytic EC-number assignments. Thus, in this study, the term “negative” refers to sequences outside the target scope of catalytic EC/KO annotation transfer rather than to proteins lacking carbohydrate-related biological relevance. Applying the same clustering criteria retained 67,880 negative representatives.

Hard sequences were derived from 21 maize silage microbial metagenomes. The silage material consisted of maize collected from Dingxi, Gansu Province, China. The dataset comprised 21 metagenomic samples, including three fresh maize samples collected before ensiling and 18 ensiled samples. The 18 ensiled samples followed a 3 × 2 design consisting of three treatments and two ensiling times, with three biological replicates for each treatment × time combination. The three ensiled treatments were CK, the TR-31-labeled inoculated treatment, and the M3-6023-labeled inoculated treatment. CK was the uninoculated control and received no additive, whereas both inoculated treatments received Lacticaseibacillus paracasei at 1 × 10^5^ CFU kg^−1^ fresh matter [18,19]. The original experimental records used the labels TR-31 and M3-6023 for the two *Lacticaseibacillus paracasei*-inoculated treatment groups, but verified strain-level designations were not available. Therefore, these labels are used here only to distinguish treatment groups and are not interpreted as strain identifiers. For subsequent analyses, the six ensiled metagenomic groups were denoted CK_7d, T, M3-6023, CK_90d, TR, and M3. T and TR represented the TR-31-labelled inoculated treatment at 7 and 90 d, respectively, whereas M3-6023 and M3 represented the M3-6023-labelled inoculated treatment at 7 and 90 d, respectively. Raw paired-end Illumina reads underwent quality control, assembly using MEGAHIT v1.2.9 [20], and gene prediction using Prodigal v2.6.3 [21]. Functional annotation was performed using CAZy [5], KEGG [6], and InterProScan v5.28-67.0 [7].

Of the 4,488,161 predicted protein-coding genes, 663,739 lacked effective annotations across all queried resources and were defined as strict hard sequences, whereas 432,307 retained only low-information annotations, such as domains of unknown function, and were defined as extended hard sequences. These categories accounted for 14.79% and 9.63% of the gene pool, respectively. These operationally defined categories do not encompass all genes with incomplete or uncertain annotations. To limit computational cost, round-robin truncation retained 500,000 strict and 300,000 extended hard sequences. Subsequent CD-HIT clustering at 90% sequence identity and 90% alignment coverage yielded 312,161 strict and 147,004 extended representatives.

The final classifier training set comprised 863,015 representative sequences: 335,970 positive representatives, 67,880 negative representatives, and 459,165 hard-sequence representatives. The application dataset comprised the 800,000 hard sequences retained after round-robin truncation and therefore represented a computational subset of the 1,096,046 strict and extended hard sequences identified in the original gene pool. CD-HIT clustering partitioned this application dataset into 459,165 representatives and 340,835 members. The representatives were included as hard-class instances during classifier training; however, no specific carbohydrate-related functional labels were assigned to them during this stage, and their functions were inferred de novo during functional prediction. The member sequences were excluded from classifier training and inherited the predictions assigned to their corresponding representatives. Accordingly, all 800,000 selected sequences were processed by CAAC, either through direct inference of representative sequences or through representative-based inheritance. Sequences classified as negative were subsequently excluded before catalytic EC/KO-oriented annotation transfer.

### 2.3. Feature Engineering

#### 2.3.1. ESM2 Layer Selection Experiment

Protein language models encode distinct sequence representations across network layers [9,10]. To select an appropriate representation for the subsequent three-class classification task, a random sample of 5000 sequences per class was drawn from the redundancy-reduced dataset. Hidden states were extracted from layers 5, 10, 15, 20, 25, 30, and 33 of ESM2-650M [10]. [CLS]-token extraction, mean pooling, and max pooling were applied to generate 1280-dimensional fixed-length representations. Logistic regression (LR) and support vector machine (SVM) classifiers were evaluated using an 80:20 stratified train–test split, with F1-macro as the primary performance metric. This experiment was conducted solely to select the ESM2 layer and pooling strategy; the LR and SVM models fitted during this experiment were not reused in subsequent CAAC-Classifier training.

#### 2.3.2. Feature Extraction and Fusion

Based on the layer-selection results, mean pooling was applied to the layer 25 hidden states of ESM2-650M [10] to generate a 1280-dimensional embedding for each protein sequence. For each hard-sequence representative, the five nearest upstream and five nearest downstream genes were sought based on gene coordinates on the assembled contigs. Genomic neighborhoods were defined by physical proximity on assembled contigs and were not further constrained by strand orientation or predicted operon boundaries. This design was used because many metagenomic contigs were short or fragmented, and reliable operon delineation was not available without transcriptomic or experimentally supported regulatory information. The resulting context representation was therefore intended to capture coarse local-neighborhood composition rather than exact operon-level organization. The DNA sequences of the neighboring genes were encoded using normalized k-mer frequencies (k = 3, 4, and 5) and projected into a 128-dimensional genomic-context representation using random projection. The k-mer lengths of 3, 4, and 5 were selected to capture short-range nucleotide-composition patterns from neighboring genes while limiting feature sparsity and dimensionality in fragmented metagenomic contigs. Shorter k-mers provide broad compositional information, whereas longer k-mers may become sparse and less stable for short or incomplete neighboring genes. Therefore, the combined k = 3–5 representation was used as a compact local-neighborhood descriptor rather than as an exhaustive sequence-context model. Positive and negative reference sequences lacked associated contig-position and neighborhood information and were therefore assigned 128-dimensional zero vectors. Hard-sequence representatives for which genomic context could not be recovered were also assigned 128-dimensional zero vectors and were classified using their ESM2 embeddings alone. The 1280-dimensional protein embedding and the 128-dimensional context vector were concatenated to produce the final 1408-dimensional input representation. Genomic-context features were successfully recovered for 93.7% of hard-sequence representatives, including 94.1% of strict hard representatives and 92.9% of extended hard representatives. Most extraction failures resulted from short or fragmented contigs that lacked sufficient neighboring genes.

### 2.4. Model Architecture and Prediction

#### 2.4.1. Classifier Architecture

The CAAC-Classifier received a 1408-dimensional fused representation comprising a 1280-dimensional ESM2 embedding and a 128-dimensional genomic-context vector. Because the two feature channels differed in dimensionality and information content, an attention-based classifier was used to learn a joint representation from the fused features [22,23] (Figure 1). The fused input was first projected into a 512-dimensional hidden space and then processed by an eight-head self-attention module followed by a feed-forward network. Residual connections and layer normalization were applied to stabilize model training, and the output layer produced probabilities for the negative, positive, and hard classes.

Model optimization used AdamW with a learning rate of 5 × 10^−4^ and a weight decay of 1 × 10^−5^. Dropout and attention dropout were set to 0.3 and 0.1, respectively. Model performance was evaluated using five-fold stratified cross-validation, with F1-macro as the primary evaluation metric.

#### 2.4.2. Prediction and Annotation Transfer

The prediction and annotation-transfer procedure comprised three stages. First, for each query sequence, the top 50 candidate neighbors were retrieved from the positive reference library using the inner-product similarity of their ESM2 representations [10]. Second, the CAAC-Classifier assigned each query to the negative, positive, or hard class. The classifier served primarily as a negative-class exclusion gate rather than as the final functional annotator. Sequences predicted as negative were excluded from catalytic EC/KO-oriented annotation transfer and downstream analyses, whereas sequences predicted as positive or hard proceeded to neighbor-based functional voting. Under this operational definition, negative-class prediction did not imply absence of all carbohydrate-associated biological roles. A hard-class prediction indicated similarity to the annotation-poor class used during classifier training and did not itself constitute a carbohydrate-related functional assignment. Third, for each retained query, neighbors carrying at least one CAZy-family or EC-number annotation were retained, and the top five annotated neighbors were used for consensus voting. CAZy-family assignment required at least 40% agreement among the selected neighbors, whereas EC-number assignment was determined according to annotation specificity and voting frequency.

Annotation confidence was calculated from neighbor consensus and ESM2-based neighbor similarity. Consistency was defined as the proportion of the modal functional label among the top five annotated neighbors. ExcessSim quantified the scaled excess of the mean neighbor-similarity score above a fixed empirical centering constant of 0.90. The two components were combined by weighted summation to obtain the confidence score:

(1) $$Confidence=W_{c}\cdot Consistenc\;y+W_{e}\cdot ExcessSim$$

The weights Wc and We were selected by evaluating 15 combinations under the constraint Wc + We ≤ 1.0 (Table S1A). Because performance was stable across the evaluated settings with a nonzero similarity contribution, Wc = 0.8 and We = 0.2 were selected to place greater emphasis on agreement among multiple retrieved neighbors while retaining neighbor similarity as a correction term. The neighbor-similarity baseline was evaluated separately using empirical centering constants of 0.85, 0.90, and 0.95 (Table S1B). The 0.85 and 0.90 settings produced nearly identical Tier 1–Tier 3 distributions, whereas the stricter 0.95 setting shifted a substantial fraction of predictions to Tier Low. The number of all integrable KO–EC features remained similar across the evaluated baselines. Therefore, 0.90 was retained as an empirical centering constant that preserved stable Tier 1/Tier 2 assignment while avoiding excessive reassignment to insufficient-confidence predictions. Because this baseline was empirically selected rather than biologically defined, confidence scores were interpreted as relative ranking measures for confidence-tier assignment rather than calibrated probabilities.

Predictions were stratified into four confidence tiers according to the confidence score:

(2) $$Tier=\left\{\begin{array}{cc} Tier\;1\;(high\;confidence) & Confidence\geq 0.60\\ Tier\;2\;(medium\;confidence) & 0.40\leq Confidence<0.60\\ Tier\;3\;(low\;confidence) & 0.25\leq Confidence<0.40\\ Tier\;Low\;(very\;low\;confidence) & Confidence<0.25 \end{array}\right.$$

Tier-specific performance was evaluated using held-out predictions from five-fold cross-validation (Table S2). The resulting precision estimates were used to establish the relative ordering of the confidence tiers rather than to equate cross-validation precision with independent functional-annotation accuracy. Tier 1, Tier 2, Tier 3, and Tier Low were therefore designated as high-, medium-, low-, and insufficient-confidence assignments, respectively. Confidence scores and tier assignments were calculated only for sequences classified as positive or hard; sequences classified as negative were excluded before neighbor voting and tier assignment. Tier 1 and Tier 2 assignments formed the candidate integration set, whereas final inclusion additionally required a valid functional annotation, successful EC-to-KO mapping, and satisfaction of the downstream integration criteria described in Section 2.5. Tier 3 and Tier Low assignments were excluded from downstream analyses.

#### 2.4.3. Database-Derived Temporal Benchmark of Functional-Annotation Transfer

To evaluate the temporal robustness of CAAC-mediated functional-annotation transfer, a database-derived benchmark was constructed using the July 2025 CAZy release. The CAZy-derived positive sequences used for model development originated from the database snapshot dated 31 July 2020. Benchmark candidates were restricted to GH, GT, and CE family sequences with explicit EC-number mappings.

A family-stratified sampling strategy was used to construct a 5000-sequence benchmark set. A maximum of 50 sequences was sampled per family, with priority given to families underrepresented in the training data, thereby producing a family composition distinct from that of the training set. CAZy-family and EC-number labels were masked to simulate annotation-poor query sequences, and the sequences were processed through the complete CAAC pipeline, including neighbor retrieval, classifier prediction, functional voting, confidence-tier assignment, and EC-to-KO mapping. Predicted annotations were compared with the masked CAZy-derived reference labels using four annotation-transfer consistency metrics. CAZy family any-match consistency required overlap between the predicted and reference family sets. CAZy Top-1 consistency required the first predicted family to match any member of the reference family set. EC exact-match consistency required complete agreement between the predicted and reference EC numbers, whereas EC level-3 consistency required agreement in the first three EC-number fields. Performance was additionally stratified by confidence tier.

This benchmark was designed to assess temporal consistency of annotation transfer within a later CAZy release rather than to verify enzyme activity experimentally. Therefore, the resulting metrics were used to evaluate the reliability of CAAC-derived annotation transfer and confidence-tier ranking, not as direct evidence of biochemical function in the target silage metagenomes.

### 2.5. Downstream Analysis of CAAC-Supplemented Annotations

#### 2.5.1. Pathway Coverage and Co-Occurrence Network Analysis

Accepted Tier 1 and Tier 2 assignments with valid EC-to-KO mappings were matched to genes using the combined Sample and GeneID identifiers and integrated with the corresponding original KEGG annotations. KO–EC mapping relationships were parsed from the KEGG ko00001.tsv file. When a predicted EC number mapped to multiple KOs, the first exact match returned by the mapping procedure was retained; when no exact match was available, hierarchical EC-prefix matching was applied progressively. Pathway coverage before supplementation was calculated from the original annotation set generated using CAZy, KEGG, and InterProScan, whereas post-supplementation coverage was calculated from the merged annotation table. The difference between the two annotation sets represented the coverage gain attributable to CAAC.

Pathway co-occurrence networks were constructed from pathway-abundance matrices for the 21 metagenomes before and after CAAC supplementation. These networks were used as exploratory, annotation-derived summaries of pathway-abundance covariance and were not intended to infer biochemical interactions, regulatory coupling, or causal metabolic relationships. Gene-level TPM values were obtained from salmon output, and genes with TPM < 1.0 were excluded. For each sample, pathway abundance was calculated by summing the TPM values of genes assigned to the corresponding pathway. Pairwise Spearman’s rank correlations were calculated across the samples, with at least five common valid samples required for each pathway pair. Multiple-testing correction was performed using the Benjamini–Hochberg procedure [24,25]. Network edges were retained when |ρ| ≥ 0.70 and the Benjamini–Hochberg-adjusted *p*-value was ≤0.05. Both positive and negative correlations satisfying these criteria were retained. Network properties included the numbers of nodes and edges, network density, degree and betweenness centrality, clustering coefficient, and modularity-based community structure.

Newly detectable annotation-derived correlations were defined as pathway pairs that met the edge-retention criteria only after CAAC supplementation. Enhanced correlations were defined as edges retained in both the original and CAAC-supplemented networks for which the absolute correlation strength increased by more than 0.10, that is, |ρ_after_| − |ρ_before_| > 0.10. Enhanced correlations therefore represented strengthened pre-existing relationships rather than newly detected edges. Newly detectable and enhanced correlations were collectively termed annotation-dependent edge differences associated with CAAC supplementation.

#### 2.5.2. Silage-Relevance Filtering and Functional-Category Summarization

The silage-focused analysis included accepted Tier 1 and Tier 2 CAAC assignments that satisfied the annotation-validity and EC-to-KO mapping criteria. Silage relevance was evaluated by case-insensitive rule-based matching against a combined annotation string containing the KO identifier, predicted EC number, KO description, KEGG pathway name, and pathway category. Each record was assigned to one of four initial classes: direct silage carbohydrate-related (Direct_silage_carbohydrate), indirect fermentation-related (Indirect_fermentation_related), putative glycan-related annotations requiring cautious interpretation (Putative_glycan_related_caution), or Other. Direct records included terms related to fermentable sugars, starch, cellulose or cellobiose, hemicellulose or xylan, pectin, glycoside hydrolases, glycolysis, and major fermentation products. Indirect records included terms related to glycans, amino or nucleotide sugars, peptidoglycan, biofilm formation, transport, stress responses, redox processes, acid resistance, and regulation. Cautionary records included glycosphingolipid-, mucin-, keratan-, chondroitin-, and glycosaminoglycan-related annotations.

Records assigned to the direct, indirect, or cautionary classes were retained, whereas records assigned to Other were excluded. Cautionary glycan-related annotations were analyzed separately and were not treated as core evidence for silage carbohydrate metabolism. Predictions were matched to their source samples using the combined Sample and GeneID identifiers. During annotation supplementation, CAAC assignments associated with genes that already carried complete KEGG annotations were not added again. Retained records were represented at the KO–EC level and deduplicated by sample, GeneID, and KO–EC feature. When duplicate assignments occurred, the assignment with the highest confidence score was retained. Sample-level abundance summaries were calculated using gene-specific TPM values.

The retained KO–EC features were subsequently assigned to 12 curated, non-exclusive functional categories for biological summarization. These categories were defined as analytical groupings rather than official KEGG modules or experimentally validated metabolic pathways. Because an individual KO–EC feature could be assigned to more than one category, category-level summaries were interpreted independently and were not combined as mutually exclusive functional totals. Complete keyword rules, category definitions, and representative KO–EC evidence are provided in Tables S3 and S4 and in the accompanying analysis code.

#### 2.5.3. Taxon-Linked Functional Profiles and Fermentation-Quality Context

CAAC-supplemented gene-level annotations were linked to their corresponding contig-level taxonomic assignments. For each taxon–category pair, TPM-weighted abundance was calculated by summing the TPM values of genes assigned to the corresponding taxon and KO–EC functional category. These profiles were used to describe the microbial-source context of CAAC-supplemented annotations across fermentation stages and inoculation treatments. Because taxon-linked TPM-weighted abundance reflects both the abundance of the assigned taxa and the abundance of their linked functional annotations, it was not interpreted as direct evidence of enzyme activity or strain-level functional capacity.

Genus- and species-level relative-abundance profiles were used to provide community-composition context. Same-treatment stage comparisons contrasted samples collected at 7 and 90 d, whereas same-time inoculation comparisons contrasted the inoculated treatments with CK at the corresponding ensiling stage. Pairwise group comparisons were performed primarily using the Mann–Whitney U test, with Welch’s *t*-test used as a supplementary raw-p screening procedure. Multiple testing was controlled using the Benjamini–Hochberg false-discovery-rate procedure. Taxon–category combinations displayed in the main figures were selected using nominal *p* < 0.05, together with effect size, biological relevance to silage carbohydrate metabolism, and consistency with the corresponding community-composition patterns. Because the displayed combinations were selected using nominal significance together with biological and compositional criteria, these patterns were treated as exploratory rather than confirmatory.

Fermentation-quality data were available as treatment-by-time summary statistics derived from four biological replicates per combination. The available table included group means, pooled standard errors of the mean, two-way ANOVA results for the effects of ensiling day, inoculation treatment, and their interaction, and Tukey’s multiple-comparison results among treatment × day combinations. The evaluated variables included pH, dry matter, lactic acid, acetic acid, the lactic acid-to-acetic acid ratio, ethanol, water-soluble carbohydrates, ammonia nitrogen, crude protein, neutral detergent fiber, and acid detergent fiber. Because replicate-level fermentation measurements were not individually matched to the metagenomic samples, these data were used only as group-level phenotypic context. No sample-level correlation or regression analysis was performed between CAAC-supplemented functional features and fermentation-quality traits. Aerobic stability was not measured and was excluded from the analysis.

### 2.6. Ablation and Baseline Evaluation

Four input configurations were evaluated to examine the contribution of the genomic-context channel. Group A used only the 1280-dimensional ESM2 embeddings. Group B concatenated each ESM2 embedding with a copied 128-dimensional segment consisting of its first 128 dimensions, producing a 1408-dimensional input that matched the complete CAAC input in dimensionality but contained no genomic-neighborhood information. Group C used the complete CAAC representation, comprising the 1280-dimensional ESM2 embedding and the corresponding 128-dimensional genomic-context vector. Group D used only the 128-dimensional context vector to characterize classification behavior in the absence of ESM2-derived sequence information. The comparison between Groups B and C controlled for total input dimensionality. However, because positive and negative reference sequences were assigned zero context vectors whereas most hard-sequence representatives had nonzero context vectors, the experiment could not fully disentangle information encoded by the genomic neighborhood from class-associated differences in context availability.

A multi-layer perceptron (MLP) and a random forest (RF) were used as conventional baseline classifiers. Each baseline model was evaluated with two feature configurations: the 1280-dimensional ESM2 embeddings alone and the 1408-dimensional ESM2-plus-context representation. All ablation and baseline models were evaluated using the same five-fold stratified cross-validation design. F1-macro was used as the primary evaluation metric, with overall accuracy and class-specific F1 scores reported as secondary metrics. Model comparisons were based on the mean and variability of the fold-specific performance estimates.

The main CAAC evaluation and the ablation workflow were conducted as separate training executions. The main CAAC model and the full-feature Group C configuration used the same classifier architecture, hyperparameters, random seed, and five-fold stratified cross-validation design. However, complete deterministic execution was not enforced for all PyTorch 2.8.0/CUDA 12.8 operations and data-loading procedures, resulting in minor differences between their performance estimates. The main CAAC result and the Group C result were therefore reported as outputs of their respective training workflows, and comparisons among Groups A–D were made only within the common ablation workflow.

## 3. Results

### 3.1. Classifier Training Set Composition and Genomic-Context Coverage

The redundancy-reduced classifier training set comprised 863,015 representative sequences (Table 1). Hard-sequence representatives collectively accounted for 53.2% of the training set, including 36.2% strict hard and 17.0% extended hard representatives.

Before round-robin truncation, 1,096,046 of the 4,488,161 predicted protein-coding genes (24.42%) met the strict or extended hard-sequence criteria. Genomic-neighborhood context was successfully recovered for 93.7% of hard-sequence representatives, including 94.1% of strict hard and 92.9% of extended hard representatives. The remaining 6.3% lacked recoverable neighborhood context, primarily because short or fragmented contigs did not contain sufficient neighboring genes. Across the complete classifier training set, recoverable genomic-context features were therefore available for 49.86% of representative sequences; positive and negative reference sequences, as well as hard-sequence representatives without recoverable context, were represented by zero context vectors.

### 3.2. ESM2 Layer and Pooling Strategy Selection

**Figure 2:**
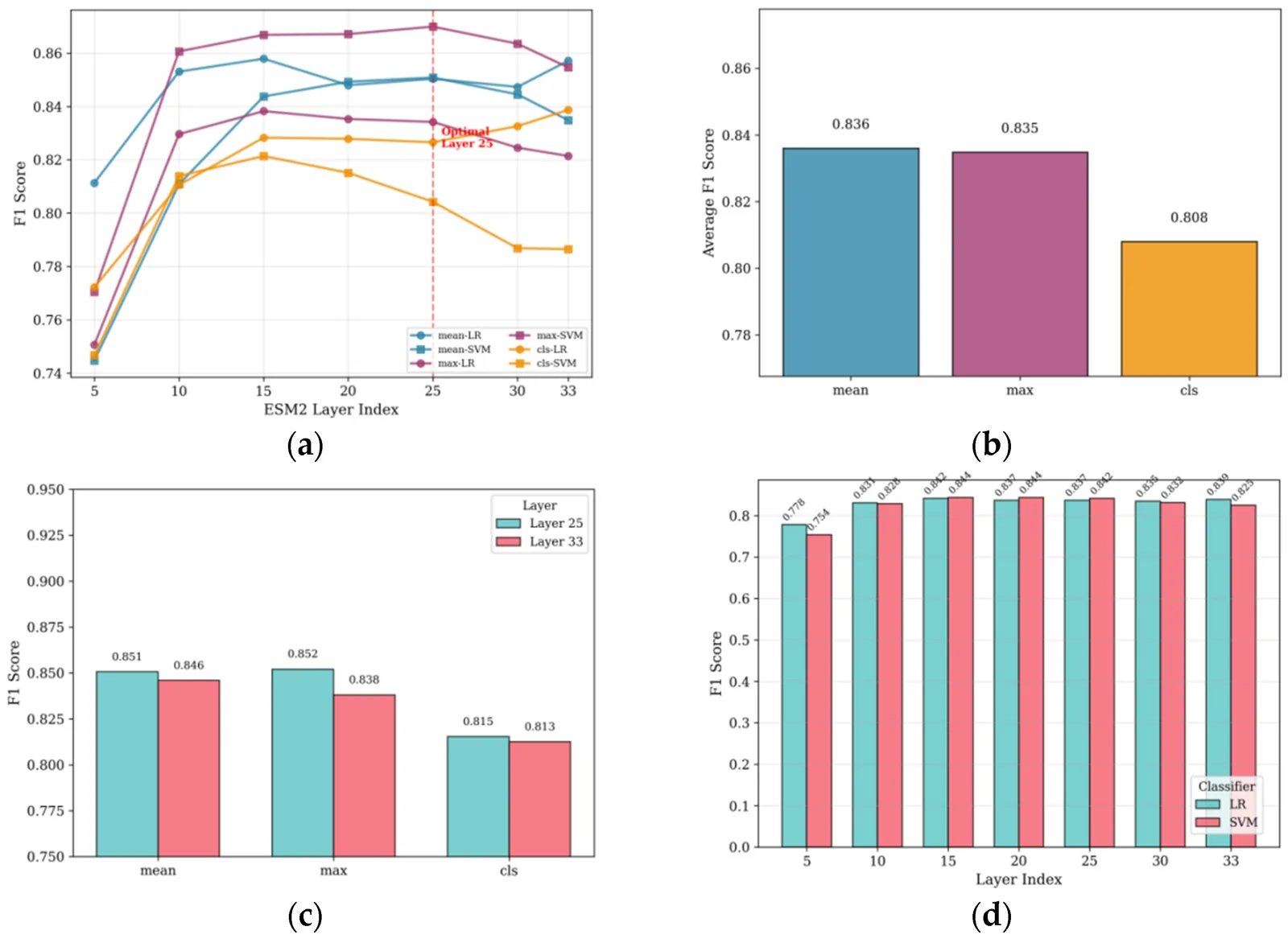
Performance comparison for ESM2 layer and pooling-strategy selection. (**a**) F1-macro values obtained using combinations of mean, max, and [CLS] pooling with logistic regression (LR) or support vector machine (SVM) across ESM2 layers 5, 10, 15, 20, 25, 30, and 33; the dashed line indicates the selected layer 25. (**b**) Mean F1-macro across the evaluated layers for mean pooling (0.836), max pooling (0.835), and [CLS] pooling (0.808). (**c**) Comparison of pooling performance between layers 25 and 33. Mean pooling achieved F1-macro values of 0.851 and 0.846 at layers 25 and 33, respectively; the corresponding values were 0.852 and 0.838 for max pooling and 0.815 and 0.813 for [CLS] pooling. (**d**) Overall comparison of LR and SVM across the evaluated layers; both classifiers exhibited similar non-monotonic trends, with LR generally achieving slightly higher F1-macro values.

Performance varied non-monotonically across the evaluated ESM2 layers and generally peaked at or near layer 25 (Figure 2). Across all evaluated layers, mean and max pooling achieved similar average F1-macro values of 0.836 and 0.835, respectively, whereas [CLS] pooling performed consistently worse, with an average F1-macro of 0.808. At layer 25, max pooling produced the highest single-configuration F1-macro of 0.852, while mean pooling achieved a nearly identical value of 0.851. At layer 33, the corresponding values decreased to 0.838 for max pooling and 0.846 for mean pooling. Logistic regression and support vector machine exhibited similar non-monotonic layer-dependent trends, although logistic regression generally achieved slightly higher performance. Because mean pooling provided the highest average performance across layers and nearly matched the best single-configuration result at layer 25, layer 25 mean-pooled embeddings were selected for subsequent feature extraction.

### 3.3. Three-Class Classification Performance of the CAAC-Classifier

**Figure 3:**
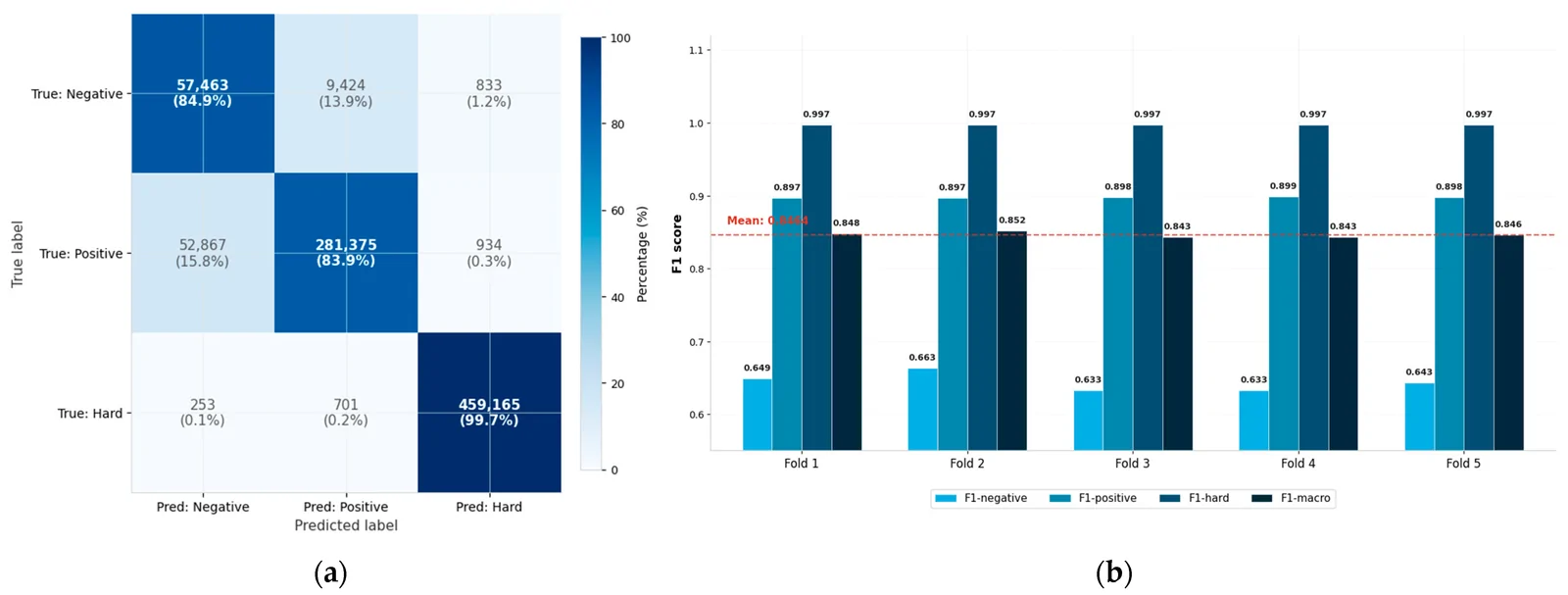
Three-class classification performance of the CAAC-Classifier. (**a**) Row-normalized confusion matrix aggregated from held-out predictions across the five cross-validation folds. (**b**) Fold-specific F1 scores for the negative, positive, and hard classes and the corresponding F1-macro values; the dashed line indicates the mean F1-macro of 0.8464.

Across five-fold cross-validation, the CAAC-Classifier achieved a mean F1-macro of 84.64 ± 0.36%, an overall accuracy of 92.46 ± 0.03%, and a weighted F1 score of 93.10 ± 0.04% (Figure 3 and Table 2). Because the three classes differed substantially in sample size, F1-macro was used as the primary summary metric, whereas overall accuracy and weighted F1 were treated as secondary measures. These results quantify discrimination among the negative, positive, and hard classes and do not represent the biological accuracy of the transferred functional annotations.

The hard class yielded the highest class-specific performance, with a recall of 99.7% and an F1 score of 99.70%. The positive and negative classes showed recalls of 83.9% and 84.9%, respectively. Most off-diagonal errors occurred between these two classes: 15.8% of true positive sequences were classified as negative, whereas 13.9% of true negative sequences were classified as positive. Consistent with this confusion pattern, the negative class had the lowest class-specific F1 score (64.42%), compared with 89.80% for the positive class. The particularly high hard-class performance was examined further through the feature-ablation analyses described in Section 3.4.

Fold-specific performance was stable, as indicated by the small standard deviations of the aggregate metrics and the narrow variation in F1-macro across folds (Figure 3b). One-vs-rest ROC analysis yielded mean AUC values of 99.97 ± 0.02% for the hard class, 98.74 ± 0.02% for the positive class, and 95.89 ± 0.07% for the negative class (Figure S1). The relatively high negative-class AUC, despite its lower F1 score, indicates stronger ranking discrimination than discrete multiclass assignment at the applied decision rule.

### 3.4. Ablation, Baseline, and Confidence-Score Calibration

Within the separate ablation workflow, the complete ESM2-plus-context configuration (Group C) achieved the highest F1-macro of 84.83%, exceeding the dimension-matched ESM2-plus-repeat control (Group B; 83.21%) by 1.62 percentage points (Table 3 and Figure S2). Replacing the repeated ESM2 dimensions with the actual genomic-context vector increased negative-class F1 from 60.40% to 64.97% and hard-class F1 from 99.49% to 99.75%, whereas positive-class F1 remained essentially unchanged (89.74% versus 89.77%). The context-only configuration (Group D) achieved an F1-macro of 61.39%, retaining a high hard-class F1 of 96.76% but yielding a negative-class F1 of 0.00%. These results show that the context channel contributed to classification under the implemented design. However, because context availability was partially associated with class identity, the observed improvement cannot be attributed exclusively to biological information encoded in the genomic neighborhood. The Group C estimate was interpreted within the ablation workflow and does not replace the main CAAC-Classifier estimate of 84.64% obtained from the separate evaluation workflow.

In the baseline comparison, the ESM2-plus-context MLP achieved an F1-macro of 84.18%, approaching the 84.64% obtained by the CAAC-Classifier, whereas the corresponding ESM2-plus-context RF achieved 70.07% (Figure S3). The difference between the MLP and CAAC-Classifier was 0.46 percentage points. Class-specific results showed that the CAAC-Classifier retained modest advantages in negative- and hard-class recognition, with F1 scores of 64.42% and 99.70%, respectively. Together with the ESM2-only ablation result of 82.16%, these findings indicate that the ESM2 embeddings provided most of the signal for the three-class task, while genomic-context integration and the attention-based classifier provided a smaller incremental improvement under the evaluated configurations.

Confidence-score performance was stable across the evaluated weight settings (Table S1). All combinations with a nonzero similarity weight (We > 0) yielded an AP of 0.9859 and a best F1 of 0.9835, whereas settings with We = 0 showed a slightly lower AP of 0.9805 while retaining the same best F1. The selected setting of Wc = 0.8 and We = 0.2, therefore, lay within a broad region of stable performance rather than representing a uniquely optimal combination. Cross-validation tier calibration yielded precision values of 97.16%, 88.52%, and 86.25% for Tiers 1, 2, and 3, respectively; no Tier Low records occurred in the calibration set (Table S2). These cross-validation estimates were used only to establish the relative ordering of the tiers and were not interpreted as estimates of annotation-transfer consistency in the temporally later CAZy benchmark. Tier 1, Tier 2, Tier 3, and Tier Low were consequently designated as high-, medium-, low-, and insufficient-confidence assignments, respectively. Only Tier 1 and Tier 2 formed the candidate integration set, and final inclusion additionally required a valid functional annotation and successful EC-to-KO mapping.

### 3.5. Application-Stage Predictions and Annotation-Transfer Consistency Benchmark

CAAC was applied to the 800,000 selected annotation-poor sequences from the 21 silage metagenomes. The CAAC-Classifier assigned 1538 sequences (0.19%) to the negative class, 5921 sequences (0.74%) to the positive class, and 792,541 sequences (99.07%) to the hard class (Table 4). The 1538 sequences classified as negative were excluded before neighbor voting and catalytic EC/KO-oriented annotation transfer, leaving 798,462 sequences classified as positive or hard for neighbor-based functional voting and confidence-tier assignment.

The predominance of hard-class predictions was expected because the application dataset had been preselected for absent or low-information annotations. A hard-class label indicated similarity to the annotation-poor class used during classifier training and did not itself constitute a carbohydrate-related functional assignment. Among the 798,462 sequences retained after negative-class exclusion, 303,505 sequences (38.01%) received Tier 1 assignments and 251,970 sequences (31.56%) received Tier 2 assignments. Together, these 555,475 sequences accounted for 69.57% of the retained prediction set and formed the predefined candidate set for annotation integration; the complete tier distribution is shown in Figure S4. After application of the functional-annotation validity and EC-to-KO mapping criteria, 545,671 predictions were retained as annotation candidates. Of these, 524,814 were associated with genes that lacked complete KEGG annotations in their source samples and were therefore eligible for sample-level annotation supplementation. The remaining 20,857 predictions were not added again because the corresponding genes already carried complete KEGG annotations.

Functional-annotation transfer consistency was assessed using 5000 label-masked GH, GT, and CE sequences sampled from the July 2025 CAZy release, whereas the CAZy-derived positive sequences used during model development originated from the database snapshot dated 31 July 2020. This database-derived temporal benchmark evaluated whether CAAC could recover masked CAZy-family and EC-number labels from a later database release after complete pipeline processing. The overall CAZy-family and EC-number consistency metrics are summarized in Table 5.

Overall, CAAC achieved a CAZy-family any-match consistency of 92.50% and a Top-1 consistency of 90.49% in this database-derived temporal benchmark. EC-number consistency was 89.23% for exact matching and 97.83% when agreement was assessed through the first three hierarchical levels. These results indicate that CAAC retained substantial annotation-transfer consistency for label-masked GH, GT, and CE sequences from a later CAZy release.

Functional-annotation transfer consistency showed a pronounced confidence-tier gradient (Table 6). Tier 1 assignments achieved a CAZy-family consistency of 94.94%, whereas the corresponding values were 45.71% for Tier 2 and 0.00% for Tier 3. These results supported the designation of Tier 1 as the high-confidence category and Tier 2 as the medium-confidence category. The substantially lower Tier 2 consistency indicates that Tier 2 assignments should be interpreted as exploratory, medium-confidence candidates rather than as predictions equivalent to Tier 1. Tier 1 and Tier 2 assignments remained subject to functional-annotation validity, EC-to-KO mapping, and sample-level supplementation criteria before downstream integration.

Because the validation set comprised known GH, GT, and CE sequences with EC mappings, these results evaluate annotation transfer within carbohydrate-active enzyme families and do not directly estimate false-positive rates among non-CAZy annotation-poor sequences in the application dataset.

### 3.6. Biological Organization of CAAC-Supplemented Silage-Related Annotations

#### 3.6.1. Pathway Coverage and Annotation-Derived Co-Occurrence Profiles After CAAC Supplementation

CAAC supplementation modestly increased mean coverage across the 564 KEGG pathways from 14.48% to 14.80%, corresponding to an absolute gain of 0.32 percentage points. Coverage increased in 64 pathways (11.3%) and remained unchanged in 500 pathways (88.7%), with the maximum gain for an individual pathway reaching 25.0 percentage points (Figure S5 and Table S5). The coverage gains were preferentially concentrated in carbohydrate metabolism, for which 25 of 47 pathways (53.2%) showed increased coverage.

Two carbohydrate metabolism pathways were newly detected after supplementation: glycosaminoglycan biosynthesis—keratan sulfate (ko00533) and mucin-type O-glycan biosynthesis (ko00512). Because both pathways belong to glycan-associated KEGG categories, they were interpreted cautiously and were not treated as core evidence for carbohydrate fermentation in silage. Among the more directly interpretable pathways, mean coverage of starch and sucrose metabolism increased from 48.52% to 57.23%, representing a gain of 8.72 percentage points, with increased coverage observed in all 21 samples. The principal interpretable contribution of CAAC was therefore the recovery of additional KO–EC components within recurrent carbohydrate-utilization pathways rather than the detection of numerous previously absent pathways (Table 7).

At the pathway co-occurrence level, CAAC supplementation changed the annotation-derived statistical association profile of pathway abundances. The total number of retained edges decreased from 13,717 before supplementation to 12,443 after supplementation, indicating that annotation supplementation did not simply increase network connectivity. Comparison of the two pathway-abundance networks identified 1087 annotation-dependent edge differences associated with CAAC supplementation, including 1062 newly detectable correlations that met the predefined edge-retention criteria only after supplementation and 25 pre-existing correlations for which the absolute correlation strength increased by more than 0.10 (|ρafter|−|ρbefore| > 0.10). Among these annotation-dependent edge differences, 623 (57.3%) involved at least one carbohydrate metabolism pathway. These results indicate that CAAC supplementation altered the detectable statistical organization of pathway-abundance profiles, particularly around carbohydrate metabolism, but the edges should be interpreted as annotation-dependent correlations rather than experimentally verified biological interactions. Detailed network-topology comparisons are provided in Figure S6 and Table S6.

#### 3.6.2. Composition and Biological Relevance of CAAC-Recovered KO–EC Features

The 545,671 Tier 1 and Tier 2 annotation candidates that passed the functional-annotation validity and EC-to-KO mapping criteria formed the input for silage-focused summarization. After source-sample matching, silage-relevance filtering, and deduplication by sample, gene identifier, and KO–EC feature, the resulting dataset contained 683,644 functional-assignment records derived from 318,740 genes and 249,970 contigs. The number of assignment records exceeded the number of sequence-level predictions because an individual sequence could contribute more than one KO–EC assignment.

Collapsing the assignment records yielded 102 unique KO–EC features. At the record level, 627,330 assignments were classified as direct silage carbohydrate-related, 31,200 as indirect fermentation-related, and 25,114 as putative glycan-related annotations requiring caution. At the feature level, the 102 KO–EC features comprised 56 direct, 25 indirect, and 21 cautionary features. Thus, most assignment records belonged to the directly silage-relevant class, whereas the cautionary glycan-related component was retained as a separately interpreted subset.

Predicted CAZy-family labels were dominated by glycosyltransferase (GT) and glycoside hydrolase (GH) families, with 408,943 GT-family mentions and 172,963 GH-family mentions. These were followed by carbohydrate-binding modules (CBM; 30,112), carbohydrate esterases (CE; 22,496), polysaccharide lyases (PL; 1622), and auxiliary activities (AA; 953). The most frequently assigned family labels were GT4, GT2, GH20, GH23, GT83, CBM50, GT35, CE4, GH24, and GT48. These counts represent predicted CAZy-family label occurrences rather than experimentally confirmed enzyme activities. They indicate that the supplemented annotations were primarily associated with glycosyl transfer, glycoside hydrolysis, and carbohydrate binding.

The five most abundant KO–EC features were glycogen phosphorylase (K00688, EC 2.4.1.1; 222,836 records), sucrose synthase (K00695, EC 2.4.1.13; 135,815 records), 1,3-β-glucan synthase (K00706, EC 2.4.1.34; 93,970 records), cellulose synthase (K00694, EC 2.4.1.12; 53,397 records), and α-amylase (K01176, EC 3.2.1.1; 39,419 records). All five features were detected in all 21 samples and together accounted for 545,437 records, corresponding to 79.8% of the silage-focused functional-assignment dataset. This distribution shows that the recovered annotation set was concentrated in a relatively small number of recurrent carbohydrate-associated functions rather than being dominated by isolated, low-frequency assignments.

In addition to these high-abundance storage-carbohydrate and glycosyltransferase-associated features, the recovered KO–EC set included annotations directly relevant to plant-derived structural polysaccharides. Representative features included acetylxylan esterase (K05972, EC 3.1.1.72), β-glucosidase (K01188, EC 3.2.1.21), xylan 1,4-β-xylosidase (K15920, EC 3.2.1.37), endoglucanase (K01179, EC 3.2.1.4), and pectate lyase (K22539, EC 4.2.2.2). Together, these features represented supplemented annotations associated with soluble sugars and storage carbohydrates, cellulose and cellobiose, xylan and hemicellulose, and pectin and glucuronate (Table 8). The biological interpretability gained through CAAC therefore extended beyond pathway-level coverage to specific enzyme-level components relevant to carbohydrate transformation during ensiling.

Representative taxonomic assignments for the recovered KO–EC features included lactic acid bacteria and other silage-associated taxa, such as *Lactiplantibacillus plantarum*, *Levilactobacillus brevis*, *Pediococcus pentosaceus*, *Lentilactobacillus buchneri*, *Loigolactobacillus coryniformis*, *Weissella cibaria*, and *Pantoea agglomerans*. These assignments were used to describe the microbial-source context of the supplemented annotations and were not interpreted as validation of enzyme activity or strain-level functional capacity.

The 102 KO–EC features were subsequently organized into 12 curated, non-exclusive silage-related functional categories for downstream summarization (Table S3), with representative supporting evidence provided in Table S4. These categories were used as interpretive groupings rather than as mutually exclusive pathways or experimentally validated metabolic modules.

#### 3.6.3. Case-Study-Based Biological Interpretation of CAAC-Supplemented Functional Profiles

To evaluate whether CAAC supplementation refined biological interpretation beyond increasing annotation counts, an exploratory case study compared original and CAAC-supplemented carbohydrate-utilization profiles (Table 9). The comparison focused on soluble-carbohydrate utilization and plant-derived structural-polysaccharide-associated microbial potential. At 7 d, the original annotation profile showed only a weak pooled inoculation-associated pattern, with Pre-CAAC log2FC values of −0.001 for starch/sucrose/oligosaccharide utilization and −0.028 for direct carbohydrate transformation. After CAAC supplementation, the corresponding values shifted to −0.136 and −0.126, respectively, indicating a clearer early inoculation-associated functional-potential signal. At 90 d, the same direction was retained but attenuated after supplementation, suggesting refinement rather than uniform amplification of the original interpretation.

CAAC also expanded enzyme-level evidence for cellulose/cellobiose-, xylan/hemicellulose-, and pectin/glucuronate-associated microbial potential. Representative features included K01179, K01188, and K00702 for cellulose/cellobiose-associated functions; K05972, K15920, and K01181 for xylan/hemicellulose-associated functions; and K01051 and K22539 for pectin/glucuronate-associated functions (Table S9). These additions shifted the interpretation from a broad carbohydrate-metabolism profile to a more enzyme-resolved description of soluble-carbohydrate turnover and plant-derived structural-polysaccharide substrate-utilization potential. Because the comparison was based on computational annotation profiles and group-level fermentation measurements, the case study was interpreted as annotation-level refinement of functional potential rather than evidence of enzyme activity, substrate degradation, or causal effects on fermentation quality.

**Figure 4:**
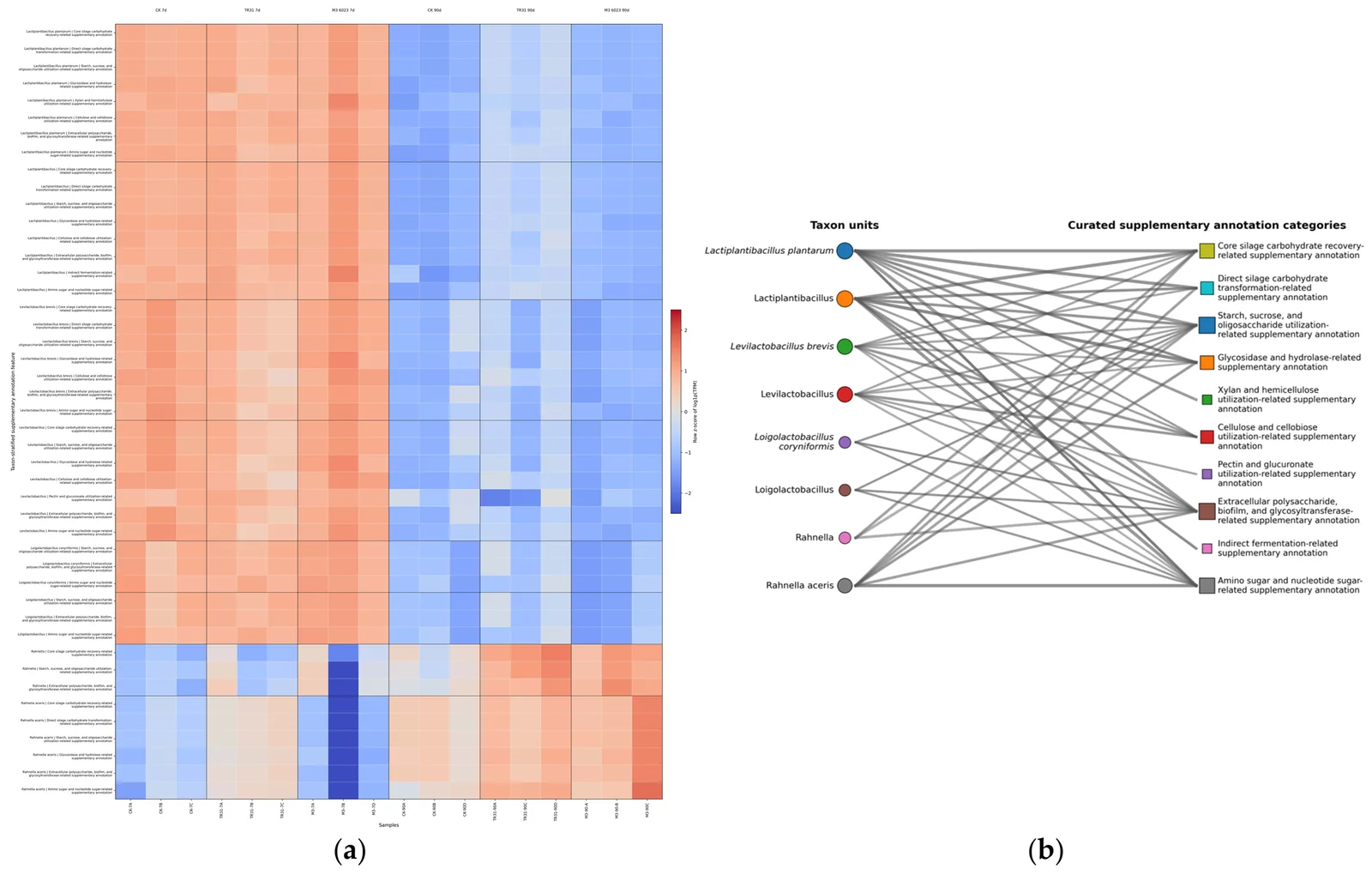
Taxon-linked profiles of CAAC-supplemented silage-related functional categories. (**a**) Heatmap of selected taxon–category pairs across individual metagenomic samples. Rows represent taxon–category pairs, columns represent samples grouped by treatment and ensiling day. (**b**) Bipartite network linking taxonomic units to curated functional categories. Edges denote the displayed taxon–category links and should not be interpreted as biochemical interactions or causal functional relationships.

Taxon-linked profiles were used to characterize the microbial-source context of CAAC-supplemented annotations across ensiling stages and inoculation treatments (Figure 4). Because TPM-weighted taxon–category abundance depends on both the abundance of the assigned taxon and the abundance of its linked functional assignments, these profiles were interpreted as compositional and ecological context rather than as direct evidence of enzyme activity or strain-level gene content. The taxon–category pairs displayed in the main figures were selected using nominal significance together with effect size, biological relevance, and consistency with the corresponding community-composition profiles; they were therefore interpreted as exploratory patterns.

CAAC-supplemented carbohydrate-related categories were linked to multiple lactic acid bacteria and other silage-associated taxa, including *Lactiplantibacillus plantarum*, *Levilactobacillus brevis*, *Loigolactobacillus coryniformis*, *Lentilactobacillus buchneri*, *Pediococcus pentosaceus*, *Weissella cibaria*, and *Rahnella aceris* (Figure 4a). TPM-weighted abundances of taxon–category pairs linked to *Lactiplantibacillus plantarum*, *Levilactobacillus brevis*, and *Loigolactobacillus coryniformis* were generally higher at 7 than at 90 d. In contrast, several pairs linked to *Lentilactobacillus buchneri* and to *Rahnella*, including *Rahnella aceris*, were more prominent at 90 d, particularly in the TR-31 and M3-6023 treatments. The taxon–category network further showed that the supplemented annotations were linked to multiple microbial taxa rather than being restricted to a single inoculant-associated taxon (Figure 4b).

**Figure 5:**
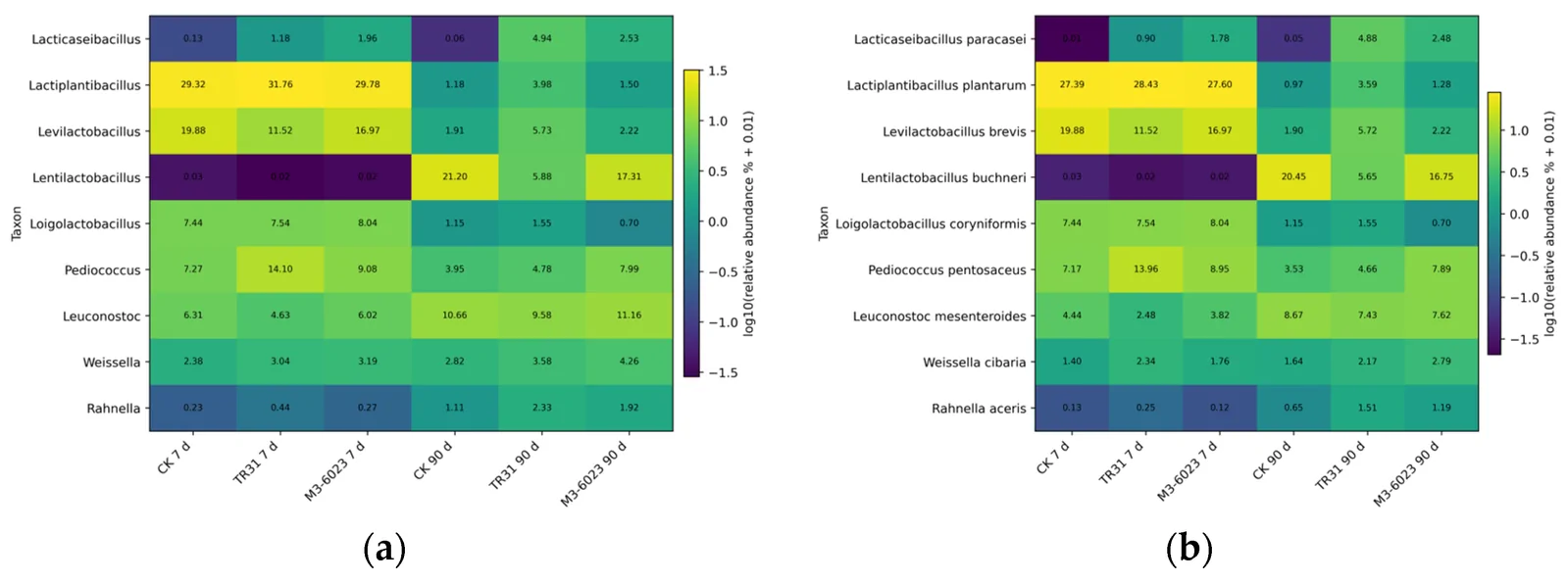
Group-level relative-abundance profiles of key silage-associated taxa. (**a**) Genus-level and (**b**) species-level heatmaps across CK, TR-31, and M3-6023 treatments at 7 and 90 d.

Community-composition profiles were consistent with the stage-associated taxon-linked patterns (Figure 5). Lactiplantibacillus and *Levilactobacillus* were prominent at 7 d, whereas *Lentilactobacillus* increased markedly at 90 d. *Lacticaseibacillus* was more abundant in the inoculated treatments and reached 4.94% in TR-31 at 90 d. Complete same-treatment stage comparisons and same-time inoculation comparisons are provided in Figures S7 and S8, respectively.

At the species level, *Lactiplantibacillus plantarum* decreased from 27.39–28.43% at 7 d to 0.97–3.59% at 90 d. *Levilactobacillus brevis* decreased from 11.52–19.88% to 1.90–5.72%, and *Loigolactobacillus coryniformis* decreased from 7.44–8.04% to 0.70–1.55%. In contrast, *Lentilactobacillus buchneri* increased from 0.02–0.03% at 7 d to 5.65–20.45% at 90 d (Figure 5b). These community-level changes provided compositional context for the taxon-linked functional profiles but were not used as independent validation of the CAAC assignments.

The inoculant species, *Lacticaseibacillus paracasei*, also showed a clear treatment-associated pattern. Its group-mean relative abundance at 7 d was 0.01% in CK, 0.90% in TR-31, and 1.78% in M3-6023, whereas the corresponding values at 90 d were 0.05%, 4.88%, and 2.48%, respectively. Group-mean TPM-weighted abundances of CAAC-supplemented annotations linked to *Lacticaseibacillus paracasei*, including starch/sucrose-, cellulose/cellobiose-, xylan/hemicellulose-, glycoside-hydrolase-, and plant structural-polysaccharide-associated categories, were also higher in the inoculated treatments than in CK (Table S7). This concordance provides inoculation-associated microbial-source context for the supplemented annotations but does not establish that the corresponding genes originated specifically from the inoculated treatment sources represented by the TR-31 and M3-6023 labels.

Group-level fermentation characteristics provided additional phenotypic context for these taxonomic and functional patterns (Table S8). The available table summarized four biological replicates per treatment × day combination. Two-way ANOVA identified significant treatment × day interactions for pH, lactic acid, ethanol, water-soluble carbohydrates, crude protein, neutral detergent fiber, and acid detergent fiber. At day 7, both pH and lactic acid showed treatment-associated differences, and significant treatment effects and treatment × ensiling–day interactions were detected for both variables (Table S8). Because the fermentation measurements were not individually matched to the metagenomic samples, no sample-level correlation or regression analysis was performed between fermentation traits and CAAC-supplemented functional features. The fermentation results were therefore interpreted as group-level experimental context rather than as direct validation of individual KO–EC assignments.

## 4. Discussion

### 4.1. Annotation Gaps and the Role of CAAC

Approximately 24.42% of the predicted protein-coding genes met the predefined strict or extended hard-sequence criteria, indicating a substantial functional-annotation gap in the 21 silage metagenomes. Similar deficits have been reported in other silage systems, in which diverse or poorly characterized microbial lineages are underrepresented in reference databases and frequently remain unresolved by conventional annotation pipelines [1,3]. Taken together, these observations suggest that annotation gaps in silage metagenomes likely arise from both the biological diversity of fermentation communities and the incomplete coverage of existing reference resources.

Culture-dependent characterization and taxonomic inference provide important biological context but are not scalable, sequence-specific solutions for assigning functions to hundreds of thousands of divergent proteins [1,3]. CAAC addresses this gap as a complementary annotation-completion framework that integrates protein-sequence representations, genomic-neighborhood information, negative-class exclusion, neighbor voting, and confidence-tiered annotation transfer. Only Tier 1 and Tier 2 assignments that passed the functional-annotation validity and EC-to-KO mapping checks were considered for integration, and assignments linked to genes with complete KEGG annotations in the corresponding source samples were not added again. CAAC is therefore intended to extend conventional annotation coverage under explicit confidence and acceptance criteria, rather than to replace homology-based annotation or experimental validation.

### 4.2. Model Performance, Feature Contributions, and Design Constraints

The ESM2 layer-selection results indicated that representations useful for the three-class classification task were not confined to the final model layer. Mean-pooled embeddings from layer 25 slightly outperformed the corresponding layer 33 representation, suggesting that non-final ESM2 representations can retain task-relevant sequence information [9,10]. This result should not be interpreted as evidence that layer 25 is universally optimal, because layer preference may vary across protein families, prediction targets, and environmental datasets. Using the selected layer 25 mean-pooled representation, the CAAC-Classifier achieved an F1-macro of 84.64% for classification of negative, positive, and hard sequences. This value quantifies performance on the three-class filtering task and does not represent the biological accuracy of the subsequent functional assignments.

Genomic-neighborhood information provided a computationally tractable complement to the protein embeddings. Functionally coupled prokaryotic genes frequently exhibit conserved co-localization [11,12], although fragmented metagenomic assemblies restrict the recovery of complete gene neighborhoods [14]. Genomic-context features were recovered for 93.7% of hard-sequence representatives, whereas the remaining 6.3% lacked recoverable neighborhood information and were represented using their ESM2 embeddings with zero context vectors. Within the separate ablation workflow, the complete ESM2-plus-context configuration achieved an F1-macro of 84.83%, compared with 83.21% for the dimension-matched ESM2-plus-repeat control. This 1.62-percentage-point difference suggested that increased input dimensionality alone was unlikely to explain all of the observed improvement.

Interpretation of this improvement is nevertheless constrained by the construction of the context channel. The context-only configuration retained a hard-class F1 of 96.76% but yielded a negative-class F1 of 0.00%. Positive and negative reference sequences were represented by zero context vectors, whereas most hard-sequence representatives received nonzero genomic-context vectors. Context presence or absence therefore constituted a class-associated signal that could be exploited independently of the biological content of the neighboring genes. Accordingly, the current ablation results demonstrate that the implemented context channel was discriminative, but they do not establish that genomic-neighborhood content alone provided an independent, biologically informative contribution. The difference between Groups B and C should therefore be interpreted as evidence for the utility of the complete context-channel implementation rather than as definitive isolation of neighborhood-content effects.

Baseline comparisons further indicated that the ESM2 embeddings contained most of the discriminative signal for the three-class task. The ESM2-plus-context MLP achieved an F1-macro of 84.18%, which was only 0.46 percentage points below the 84.64% obtained by the CAAC-Classifier, whereas the corresponding random forest model achieved 70.07%. The attention-based classifier retained modest advantages in negative- and hard-class recognition, but the small overall difference relative to the MLP does not demonstrate substantial architectural superiority. The principal methodological contribution of CAAC therefore lies in the coordinated integration of protein representations, genomic-context features, negative-class filtering, neighbor-based functional voting, confidence scoring, and annotation-transfer criteria rather than in the classifier architecture alone.

### 4.3. Biological Interpretation of CAAC-Supplemented Annotations in Silage

The principal interpretive gain provided by CAAC was observed at the enzyme and functional-feature levels rather than as widespread detection of new pathways. Although the mean coverage increase across all KEGG pathways was modest, supplementation recovered previously unrepresented KO–EC components within recurrent carbohydrate-utilization pathways, especially those related to starch and sucrose, cellulose and cellobiose, xylan and hemicellulose, and pectin and glucuronate metabolism. These predicted enzyme assignments provide a basis for interpreting carbohydrate-utilization potential during ensiling, but they do not demonstrate that the corresponding reactions were active in the samples.

Taxon-linked profiles provided microbial-source context for the supplemented annotations. Exploratory comparisons indicated that the TPM-weighted abundances of profiles linked to *Lactiplantibacillus plantarum*, *Levilactobacillus brevis*, and *Loigolactobacillus coryniformis* were generally higher during early fermentation, whereas several profiles linked to *Lentilactobacillus buchneri* and *Rahnella*, including *Rahnella aceris*, were more prominent at 90 d. The late-stage increase in *Lentilactobacillus buchneri*-linked annotation abundance was consistent with the reported association of this species with mature silage and heterofermentative fermentation [26,27,28,29,30,31,32,33,34]. However, aerobic stability was not measured, and this pattern cannot be interpreted as evidence that aerobic stability was improved. Similarly, late-stage *Rahnella*-linked pectin/glucuronate- and hemicellulose-associated annotations provide ecological and microbial-source context but do not demonstrate experimentally confirmed polysaccharide degradation.

The exploratory case study further showed that CAAC refined, rather than merely enlarged, the functional profile. Early-stage inoculation-associated differences in soluble-carbohydrate utilization were weak in the original profile but became clearer after supplementation, supported by KO–EC features related to glycogen phosphorylase, sucrose synthase, and alpha-amylase. At 90 d, the same direction was retained but attenuated, indicating that CAAC did not uniformly amplify all treatment-associated signals. The additional cellulose/cellobiose-, xylan/hemicellulose-, and pectin/glucuronate-associated features further support enzyme-resolved interpretation of microbial substrate-utilization potential, while remaining computational predictions rather than measured enzyme activity or causal fermentation mechanisms.

An inoculation-associated pattern was also observed for *Lacticaseibacillus paracasei*. Both the group-mean relative abundance of *Lacticaseibacillus paracasei* and the TPM-weighted abundances of its linked CAAC-supplemented carbohydrate-related annotations were higher in the TR-31- and M3-6023-labeled inoculated treatments than in CK. This concordance indicates that the taxon-linked analysis captured a microbial-source pattern associated with inoculation. Nevertheless, contig-level taxonomic assignment cannot determine whether the corresponding genes originated specifically from the inoculated treatment sources represented by the TR-31 and M3-6023 labels rather than from indigenous *Lacticaseibacillus paracasei* populations. Strain-specific attribution would require additional genomic or marker-based evidence.

Group-level fermentation characteristics provided phenotypic context for interpreting the supplemented annotation patterns (Table S8). Treatment effects were detected for pH, dry matter, lactic acid, acetic acid, the lactic acid-to-acetic acid ratio, ethanol, water-soluble carbohydrates, crude protein, and neutral detergent fiber, whereas significant treatment × ensiling–day interactions were observed for pH, lactic acid, ethanol, water-soluble carbohydrates, crude protein, neutral detergent fiber, and acid detergent fiber. These results confirm that inoculation and ensiling stage altered the fermentation and substrate-conversion environment in which the taxonomic and annotation patterns were observed. However, the fermentation measurements were not individually matched to the metagenomic samples, and no sample-level correlation or regression analysis was possible. The correspondence between fermentation characteristics and CAAC-supplemented carbohydrate-related annotations should therefore be interpreted as group-level biological consistency rather than as statistical or causal evidence linking individual KO–EC assignments to changes in fermentation traits.

CAAC supplementation also altered the annotation-derived pathway co-occurrence profile. Although the total number of retained edges decreased from 13,717 before supplementation to 12,443 after supplementation, comparison of the two pathway-abundance networks identified 1087 annotation-dependent edge differences, including 1062 newly detectable correlations and 25 strengthened pre-existing correlations. Among these annotation-dependent edge differences, 623 (57.3%) involved at least one carbohydrate metabolism pathway. The simultaneous loss, appearance, and strengthening of different edges indicates a redistribution of detectable pathway-abundance associations rather than a generalized increase in network connectivity.

These network differences should be interpreted cautiously because they were derived from pathway-abundance matrices whose entries changed after annotation supplementation. Therefore, the observed edge differences may reflect improved or altered annotation completeness, changes in pathway-abundance estimation, and threshold-dependent correlation structure rather than direct biological reorganization of the microbial community. The network analysis supports the conclusion that CAAC supplementation changed the statistical representation of functional profiles, whereas experimental measurements would be required to establish pathway activity, biochemical interaction, or mechanistic coupling.

### 4.4. Limitations and Future Directions

Several limitations should be considered when interpreting the results. First, the EC-to-KO transfer step introduces mapping uncertainty because some EC numbers correspond to multiple KEGG Orthology terms. The mapping procedure may therefore influence both the identities of the supplemented KOs and the resulting pathway-coverage estimates. In addition, the confidence tiers were calibrated using the constructed training and cross-validation distributions, whereas the application dataset comprised 21 metagenomes from a single silage experiment. Performance and confidence calibration may consequently differ across forage types, fermentation conditions, microbial communities, and more divergent environmental sequences. The neighbor-similarity baseline analysis showed that the number of all integrable KO–EC features remained similar across the evaluated baselines, but this parameter should still be interpreted as an empirical centering constant rather than a calibrated biological threshold. Accordingly, confidence scores were used as relative tiering measures and not as calibrated probabilities of functional correctness.

Second, genomic-context availability was class-dependent. Positive and negative reference sequences lacked associated contig-position and neighborhood information and were therefore represented by zero context vectors, whereas most hard-sequence representatives received nonzero genomic-context vectors. This design may have contributed to the particularly high hard-class performance and limited the ability to separate biologically informative neighborhood content from the simpler signal provided by context presence or absence. In addition, the genomic-context vector represented physical gene proximity rather than strand-aware or operon-resolved neighborhood organization. Adjacent genes located on opposite strands or across fragmented contig regions may not be co-transcribed or functionally coupled. Future evaluations should use matched context availability across classes, context masking, shuffled-context controls, and strand-aware or operon-informed representations where reliable neighborhood information is available. Alternative k-mer lengths and projection dimensions were not systematically evaluated in this study. Future work should also assess alternative k-mer lengths, projection dimensions, and learned nucleotide-context encoders.

Third, because CBMs can contribute to carbohydrate binding and substrate recognition, CAAC should be interpreted as targeting catalytic or EC/KO-transferable carbohydrate-related functions rather than the full spectrum of carbohydrate-associated proteins.

Fourth, the July 2025 CAZy benchmark provided a temporally later, database-derived assessment of CAAC-mediated annotation transfer. Because the benchmark sequences and the training positives were both derived from CAZy and belonged to the same broad enzyme-family system, the benchmark primarily evaluated temporal annotation-transfer consistency within known carbohydrate-active enzyme families. It did not constitute biochemical validation of enzyme activity, nor did it directly estimate performance on non-CAZy annotation-poor sequences from the target silage metagenomes. Tier 1 performance therefore supports the use of Tier 1 as a high-confidence annotation-transfer category, whereas the lower Tier 2 performance supports its treatment as a medium-confidence, exploratory category. The transferred assignments should be interpreted as computationally inferred functional potential that requires independent biochemical, genetic, or strain-resolved verification.

Fifth, the 12 silage-related functional categories were generated through reproducible rule-based matching of KO–EC annotations, functional descriptions, pathway terms, and silage-relevance keywords. They represent non-exclusive analytical summaries rather than official KEGG modules or experimentally established metabolic pathways. Individual features could be assigned to more than one category, and category-level abundances should therefore not be summed as independent functional compartments. In addition, the putative glycan-related cautionary categories were retained for completeness but were not treated as core evidence for carbohydrate fermentation in silage.

Sixth, contig-level taxonomic assignments provided microbial-source context but did not support strain-resolved tracking. Genes assigned to *Lacticaseibacillus paracasei* could not be attributed specifically to the inoculated treatment sources represented by the TR-31 and M3-6023 labels rather than to indigenous *Lacticaseibacillus paracasei* populations. Such attribution would require additional evidence from strain-specific reference genomes or markers, competitive read mapping, SNP-based tracking, or strain-resolved metagenome-assembled genomes.

Seventh, the metagenomic sampling design included fresh material and silages collected at 7 and 90 d, without intermediate sampling between day 0 and day 7. Accordingly, the day-7 measurements were interpreted as an early-fermentation endpoint rather than as a time-resolved estimate of acidification kinetics. The available fermentation-quality data consisted of treatment-by-time summary statistics derived from four biological replicates per combination, but the replicate-level measurements were not individually matched to the 21 metagenomic samples. Consequently, no sample-level correlation or regression analysis was performed between CAAC-supplemented KO–EC features and fermentation traits. Aerobic stability was not measured and was therefore not evaluated in relation to the late-stage taxonomic or fermentation patterns. Taxon-linked profiles were therefore interpreted only as microbial-source context for the supplemented annotations.

Future work should evaluate CAAC on independent silage and other environmental metagenomic datasets. Representative Tier 1 assignments require experimental validation through heterologous expression and biochemical assays. Integrating metagenomic predictions with sample-matched metabolomic, transcriptomic, fermentation, and enzyme-activity measurements would further bridge the gap between computational inference and biological mechanism. In parallel, strain-resolved analyses and context-masking experiments are needed to distinguish inoculant-derived from indigenous populations and to quantify the independent contribution of genomic-neighborhood content.

## 5. Conclusions

CAAC was developed as a complementary framework for supplementing functional annotations of annotation-poor protein sequences in silage metagenomes by integrating ESM2 embeddings, genomic-neighborhood features, negative-class exclusion, confidence-tiered neighbor voting, and EC-to-KO mapping. In five-fold cross-validation, the CAAC-Classifier achieved an F1-macro of 84.64% for three-class classification of negative, positive, and hard sequences. A database-derived temporal benchmark using the July 2025 CAZy release showed 94.94% CAZy-family annotation-transfer consistency for Tier 1 assignments, supporting their designation as high confidence, whereas the substantially lower Tier 2 consistency supported its treatment as a medium-confidence, exploratory category.

When applied to 800,000 selected annotation-poor sequences from 21 silage metagenomes, CAAC retained 545,671 Tier 1 or Tier 2 annotation candidates that passed the functional-annotation validity and EC-to-KO mapping criteria; 524,814 of these were eligible for sample-level KEGG annotation supplementation. Silage-focused summarization yielded 102 KO–EC features repeatedly detected across the silage metagenomes, and CAAC supplementation increased coverage in 25 of 47 carbohydrate metabolism pathways. Although two carbohydrate-metabolism pathways were newly detected after supplementation, the principal interpretable gain was the recovery of enzyme-level components associated with starch and sucrose, cellulose and cellobiose, xylan and hemicellulose, and pectin and glucuronate utilization rather than the detection of numerous new pathways. Annotation-derived pathway co-occurrence comparison identified 1087 annotation-dependent edge differences associated with CAAC supplementation, of which 57.3% involved carbohydrate metabolism. Exploratory taxon-linked analyses further provided stage- and inoculation-associated microbial-source context, including higher TPM-weighted abundances of *Lacticaseibacillus paracasei*-linked annotations in the inoculated treatments.

Overall, CAAC provides a scalable, confidence-stratified strategy for supplementing conventional functional annotations in silage metagenomes. Its principal contribution is to extend enzyme-level interpretation of recurrent carbohydrate-utilization pathways while preserving explicit filtering and integration criteria. The resulting assignments represent computationally inferred functional potential rather than experimentally confirmed enzyme activity, causal pathway relationships, or strain-specific gene content. CAAC should therefore be regarded as a complement to conventional annotation and prioritization of candidate functions, and representative predictions will require biochemical and strain-resolved verification.

## Figures and Tables

**Table 1:** Training set composition.

Category	Pre-Clustering Sequences Included in the Analysis	Post-Clustering Representatives	Proportion of the Training Set
Positive sequences	2,132,926	335,970	38.9%
Negative sequences	376,080	67,880	7.9%
Strict hard sequences	500,000	312,161	36.2%
Extended hard sequences	300,000	147,004	17.0%
Total	—	863,015	100%

Note: Before round-robin truncation, the strict and extended hard-sequence pools contained 663,739 and 432,307 sequences, respectively; 500,000 strict and 300,000 extended hard sequences were retained for CD-HIT clustering. Percentages refer to the final redundancy-reduced classifier training set and do not represent the prevalence of these classes in the original metagenomic gene pool.

**Table 2:** Five-fold cross-validation performance of the CAAC-Classifier.

Statistic	F1-Macro	Accuracy	Weighted F1	Negative-Class F1	Positive-Class F1	Hard-Class F1
Mean ± SD	84.64 ± 0.36%	92.46 ± 0.03%	93.10 ± 0.04%	64.42 ± 1.23%	89.80 ± 0.45%	99.70 ± 0.08%

**Table 3:** Ablation performance of the CAAC input configurations.

Group	Input Dimension	F1-Macro	Negative-Class F1	Positive-Class F1	Hard-Class F1
A: ESM2 only	1280	82.16%	57.21%	89.81%	99.48%
B: ESM2 + Repeat	1408	83.21%	60.40%	89.74%	99.49%
C: ESM2 + Context	1408	84.83%	64.97%	89.77%	99.75%
D: Context only	128	61.39%	0.00%	87.42%	96.76%

**Table 4:** CAAC-Classifier outputs and downstream handling for the 800,000-sequence application dataset.

Predicted Class	Sequences	Proportion	Downstream Handling
Negative	1538	0.19%	Excluded before neighbor voting and catalytic EC/KO-oriented annotation transfer
Positive	5921	0.74%	Retained for neighbor-based functional voting
Hard	792,541	99.07%	Retained for neighbor-based functional voting
Total	800,000	100.00%	—

**Table 5:** CAZy-derived temporal annotation-transfer consistency benchmark using the July 2025 CAZy release.

Metric	Consistency	Description
CAZy any-match	92.50%	The predicted family set overlapped the reference family set
CAZy Top-1	90.49%	The first predicted family matched any member of the reference family set
EC exact match	89.23%	The predicted EC number exactly matched the reference EC number
EC level-3 match	97.83%	The first three EC-number fields matched the reference EC number

**Table 6:** Confidence-tier-stratified CAZy-family annotation-transfer consistency in the July 2025 CAZy benchmark.

Tier	Annotation Assignments	Proportion	CAZy-Family Consistency
Tier 1	4844	96.4%	94.94%
Tier 2	105	2.1%	45.71%
Tier 3	75	1.5%	0.00%

Note: Counts represent annotation assignments rather than unique benchmark sequences. The 5024 assignments exceeded the 5000 benchmark sequences because some sequences received multiple predicted annotations through different voting paths, and each assignment was scored and tiered separately. Tier-specific CAZy-family consistency was calculated at the annotation-assignment level. Because the benchmark set comprised known GH, GT, and CE sequences with EC mappings, these results evaluate annotation transfer within carbohydrate-active enzyme families and do not directly estimate false-positive rates among non-CAZy annotation-poor sequences in the application dataset.

**Table 7:** Representative carbohydrate metabolism pathways showing newly detected or increased coverage after CAAC supplementation.

Pathway ID	Pathway Name	Coverage Before (%)	Coverage After (%)	Mean Gain (Percentage Points)	Interpretation
ko00533	Glycosaminoglycan biosynthesis—keratan sulfate	0.00	7.82	7.82	Glycan-associated
ko00512	Mucin type O-glycan biosynthesis	0.00	9.84	9.84	Glycan-associated
ko00603	Glycosphingolipid biosynthesis—globo and isoglobo series	19.05	33.93	14.88	Glycan-associated
ko00531	Glycosaminoglycan degradation	21.90	37.14	15.24	Glycan-associated
ko00511	Other glycan degradation	38.53	48.48	9.96	Glycan-associated
ko00500	Starch and sucrose metabolism	48.52	57.23	8.72	Directly interpretable carbohydrate-utilization pathway
ko01003	Glycosyltransferases	12.03	16.06	4.03	Carbohydrate transformation/glycosyl-transfer potential
ko00053	Ascorbate and aldarate metabolism	37.94	40.06	2.12	Pectin/glucuronate-associated carbohydrate metabolism context

**Table 8:** Representative CAAC-recovered KO–EC features directly relevant to silage carbohydrate metabolism.

Feature ID	Predicted Function	Main Substrate/Process	Major Taxonomic Sources
K00688|2.4.1.1	Glycogen phosphorylase	Glycogen/storage carbohydrate mobilization	*Lactiplantibacillus plantarum*; *Levilactobacillus brevis*; *Pediococcus pentosaceus*
K00695|2.4.1.13	Sucrose synthase	Sucrose conversion	*Lactiplantibacillus plantarum*; *Levilactobacillus brevis*; *Pediococcus pentosaceus*
K01176|3.2.1.1	α-Amylase	Starch degradation	*Lactiplantibacillus plantarum*; *Levilactobacillus brevis*; *Pediococcus pentosaceus*
K01188|3.2.1.21	β-Glucosidase	β-glucosides/cellobiose-related substrates	*Lactiplantibacillus plantarum*; *Weissella cibaria*; *Pantoea agglomerans*
K05972|3.1.1.72	Acetylxylan esterase	Acetylated xylan hemicellulose	*Levilactobacillus brevis*; *Lactiplantibacillus plantarum*; *Lentilactobacillus buchneri*
K15920|3.2.1.37	Xylan 1,4-β-xylosidase	Xylan/xylo-oligosaccharides	*Lactiplantibacillus plantarum*; *Pseudocitrobacter faecalis*; *Weissella cibaria*
K01179|3.2.1.4	Endoglucanase	Cellulose/glucan substrates	*Lactiplantibacillus plantarum*; *Levilactobacillus brevis*; *Pantoea agglomerans*
K22539|4.2.2.2	Pectate lyase	Pectate/pectin-associated substrates	*Lactiplantibacillus plantarum*; *Pseudocitrobacter faecalis*; *Pantoea agglomerans*

**Table 9:** Exploratory comparison of original and CAAC-supplemented carbohydrate-utilization profiles.

Functional Aspect	Original Profile	CAAC-Supplemented Profile	Representative CAAC Features	Interpretation After CAAC
Soluble-carbohydrate potential at 7 d	log2FC = −0.001/−0.028	log2FC = −0.136/−0.126	K00688, K00695, K01176	Early inoculation-associated signal became clearer
Soluble-carbohydrate potential at 90 d	log2FC = −0.313/−0.261	log2FC = −0.135/−0.145	K00688, K00695, K01176	Direction retained but attenuated
Cellulose/cellobiose-associated potential	Low original TPM, mostly 1900–2500	CAAC accounted for approximately 90–95% of Post-CAAC TPM	K01179, K01188, K00702	Structural-polysaccharide-related potential was expanded
Xylan/hemicellulose-associated potential	Limited category-level support	K05972, K15920, and K01181 recovered	K05972, K15920, K01181	Hemicellulose-related microbial potential was refined
Pectin/glucuronate-associated potential	Present but weakly represented	K01051 TPM = 7772; K22539 records = 3161	K01051, K22539	Pectin/glucuronate-related potential was added

Note: For soluble-carbohydrate potential, the two log2FC values indicate starch/sucrose/oligosaccharide utilization and direct carbohydrate transformation, respectively. Negative log2FC values in the CK-versus-inoculated contrast indicate higher values in inoculated treatments. CAAC-supplemented features represent computationally inferred EC/KO-transferable functional potential rather than experimentally measured enzyme activity.

## Data Availability

Raw metagenomic sequencing data have been deposited in the NCBI Sequence Read Archive under project accession number PRJNA1474118. The complete CAAC source code, pretrained model weights, and analysis pipeline documentation are available at the GitHub repository https://github.com/TheSeasonForHer/ESM2_CAAC/ (accessed on 17 August 2026).

## Supplementary Materials

The following supporting information can be downloaded at: https://www.mdpi.com/article/10.3390/microorganisms14081848/s1, Figure S1: ROC curves of the CAAC-Classifier from 5-fold cross-validation; Figure S2: Ablation study results for context-aware feature integration; Figure S3: Baseline model comparison for three-class sequence classification; Figure S4: Confidence-tier distribution and score profile after negative-class exclusion; Figure S5: Pathway coverage distribution before and after backfilling; Figure S6: Annotation-derived pathway co-occurrence topology before and after CAAC supplementation; Figure S7: Same-treatment stage-associated patterns in selected taxon-linked CAAC-supplemented functional categories; Figure S8: Same-time inoculation-associated patterns in selected taxon-linked CAAC-supplemented functional categories; Table S1: Sensitivity analyses for confidence-score parameter selection. (A) Confidence-score weight ablation results. Fifteen weight combinations were evaluated under the constraint Wc + We ≤ 1.0. AP, best F1, and best threshold are reported for each combination. (B) Sensitivity of confidence-tier assignment to the neighbor-similarity baseline; Table S2: Actual precision of each tier on the cross-validation test set; Table S3: Curated non-exclusive silage-related functional categories summarized from CAAC-supplemented annotations; Table S4: Representative KO–EC evidence supporting curated category assignment; Table S5: Pathway coverage changes before and after backfilling; Table S6: Annotation-derived pathway co-occurrence topology before and after CAAC supplementation; Table S7: Group-mean TPM-weighted abundance of Lacticaseibacillus paracasei-linked CAAC-supplemented carbohydrate-related categories; Table S8: Fermentation characteristics and chemical composition of CK and Lacticaseibacillus paracasei-inoculated treatment groups after 7 and 90 days of ensiling; Table S9: Representative CAAC-supplemented KO–EC features supporting the exploratory case study.

## Abbreviations

The following abbreviations are used in this manuscript:

AA	auxiliary activities
ANOVA	analysis of variance
AP	average precision
AUC	area under the curve
CAAC	Context-Aware Annotation Completion
CAZy	Carbohydrate-Active Enzymes database
CBM	carbohydrate-binding module
CD-HIT	Cluster Database at High Identity with Tolerance
CE	carbohydrate esterase
[CLS]	classification token
CUDA	Compute Unified Device Architecture
EC	Enzyme Commission
ESM2	Evolutionary Scale Modeling 2
FFN	feed-forward network
GH	glycoside hydrolase
GT	glycosyltransferase
KEGG	Kyoto Encyclopedia of Genes and Genomes
KO	KEGG Orthology
LR	logistic regression
MLP	multilayer perceptron
NCBI	National Center for Biotechnology Information
PL	polysaccharide lyase
RF	random forest
ROC	receiver operating characteristic
SD	standard deviation
SNP	single-nucleotide polymorphism
SVM	support vector machine
TPM	transcripts per million
