# Supplementary material for: ESM2-Guided Context-Aware Annotation Completion Supplements Carbohydrate Metabolism Coverage in Silage Microbial Metagenomes
Source: Microorganisms. 2026 Aug 20;14(8):1848. doi: 10.3390/microorganisms14081848 (PMC13515629; doi:10.3390/microorganisms14081848)
Supplement: Supplementary file 1 [file microorganisms-14-01848-s001.zip › microorganisms-4494931-supplementary.pdf]

Supplementary Materials for

ESM2-Guided Context-Aware Annotation Completion Supplements Carbohydrate Metabolism Coverage in Silage Microbial Metagenomes

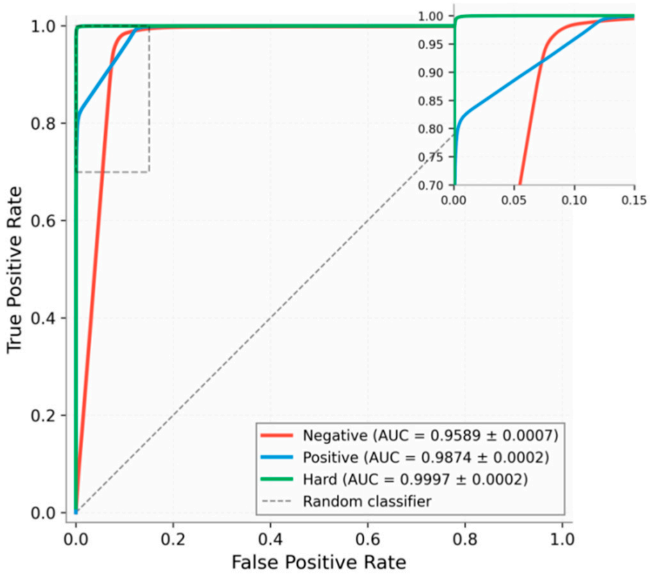

Figure S1. ROC curves of the CAAC-Classifier from 5-fold cross-validation.

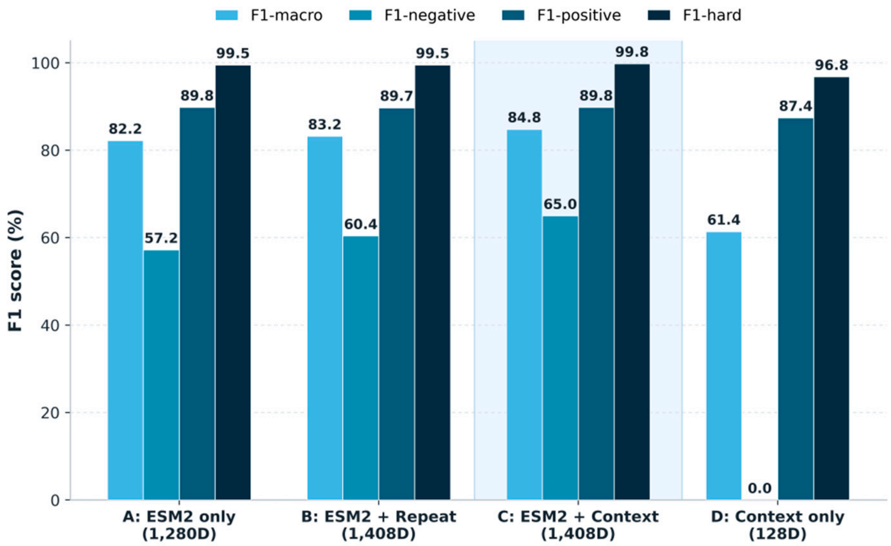

Figure S2. Ablation study results for context-aware feature integration.

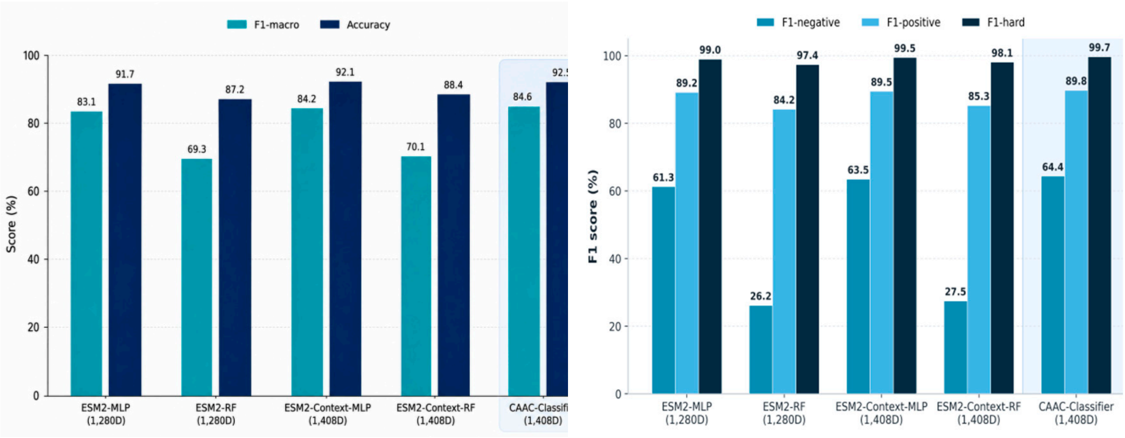

(**a**) (**b**)

Figure S3. Baseline model comparison for three-class sequence classification. (a) Overall performance comparison of F1-macro and accuracy across five models, including ESM2-MLP (1,280D), ESM2-RF (1,280D), ESM2-Context-MLP (1,408D), ESM2-Context-RF (1,408D), and CAAC-Classifier (1,408D). (b) Per-class F1 score comparison showing F1-negative, F1-positive, and F1-hard scores for each model. All results are reported as the mean of 5-fold cross-validation. The optimized MLP baseline achieved an F1-macro of 84.18%, approaching that of the CAAC-Classifier (84.64%), whereas the RF baseline (70.07%) remained substantially lower. The CAAC-Classifier retained advantages in negative-class recognition (F1-Negative 64.42%) and hard-sequence identification (F1-Hard 99.70%).

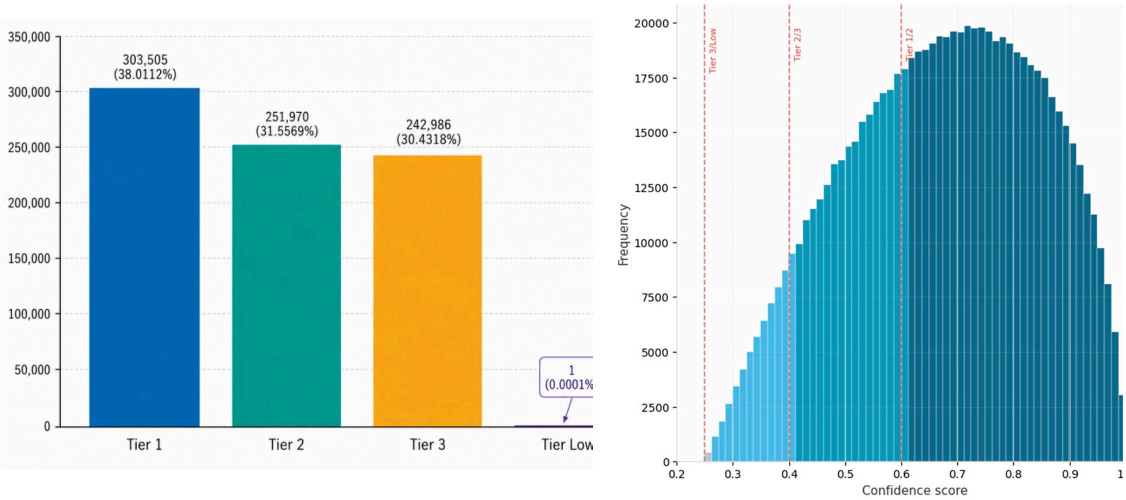

(**a**) (**b**)

Figure S4. Confidence-tier distribution and score profile after negative-class exclusion. (a) Final tier distribution among the 798,462 sequences classified as positive or hard and retained for annotation evaluation. Tier 1 contained 303,505 sequences (38.01%), Tier 2 contained 251,970 sequences (31.56%), Tier 3 contained 242,986 sequences (30.43%), and Tier Low contained one sequence (0.0001%). (b) Confidence-score distribution among the 798,462 retained sequences, with vertical dashed lines indicating the tier thresholds at 0.60 (Tier 1/Tier 2 boundary), 0.40 (Tier 2/Tier 3 boundary), and 0.25 (Tier 3/Tier Low boundary).

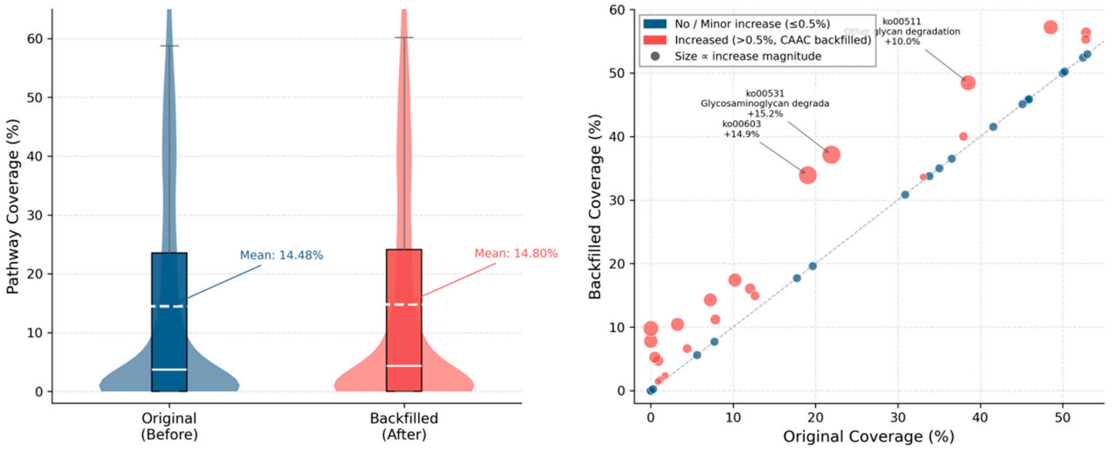

(a) (b)

Figure S5. Pathway coverage distribution before and after backfilling. (a) Overall pathway coverage distribution. The mean pathway coverage increased from 14.48% before backfilling to 14.80% after backfilling. (b) Carbohydrate metabolism pathways. Most pathways showed increased coverage after backfilling (red circles), with notable improvements including ko00531 (+15.2%), ko00603 (+14.9%), and ko00511 (+10.0%).

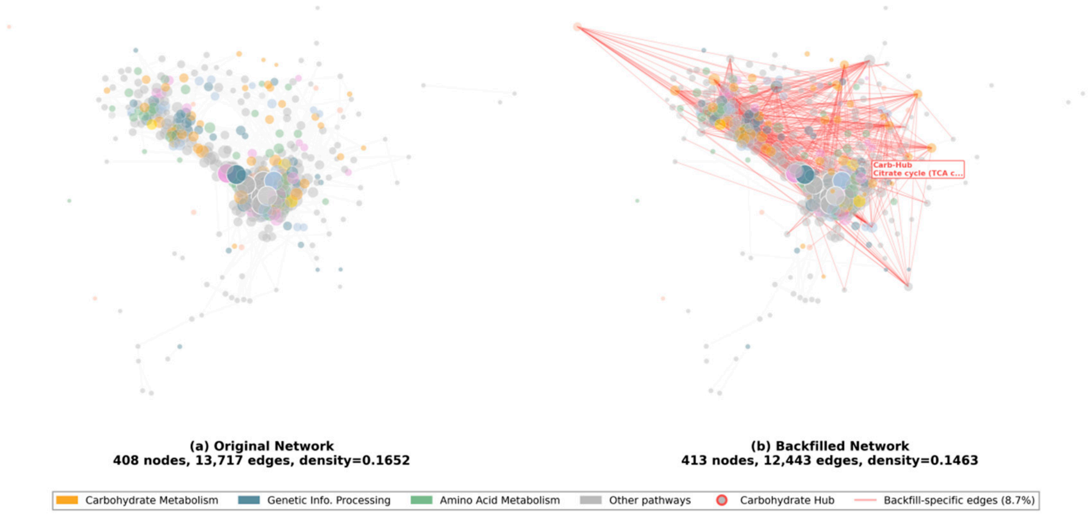

Figure S6. Annotation-derived pathway co-occurrence topology before and after CAAC supplementation. Edges were retained based on Spearman correlation thresholds applied to pathway-abundance matrices before and after annotation supplementation. Edge presence, loss, or gain reflects changes in annotation-derived pathway-abundance associations and should not be interpreted as direct biochemical interactions or causal metabolic relationships.

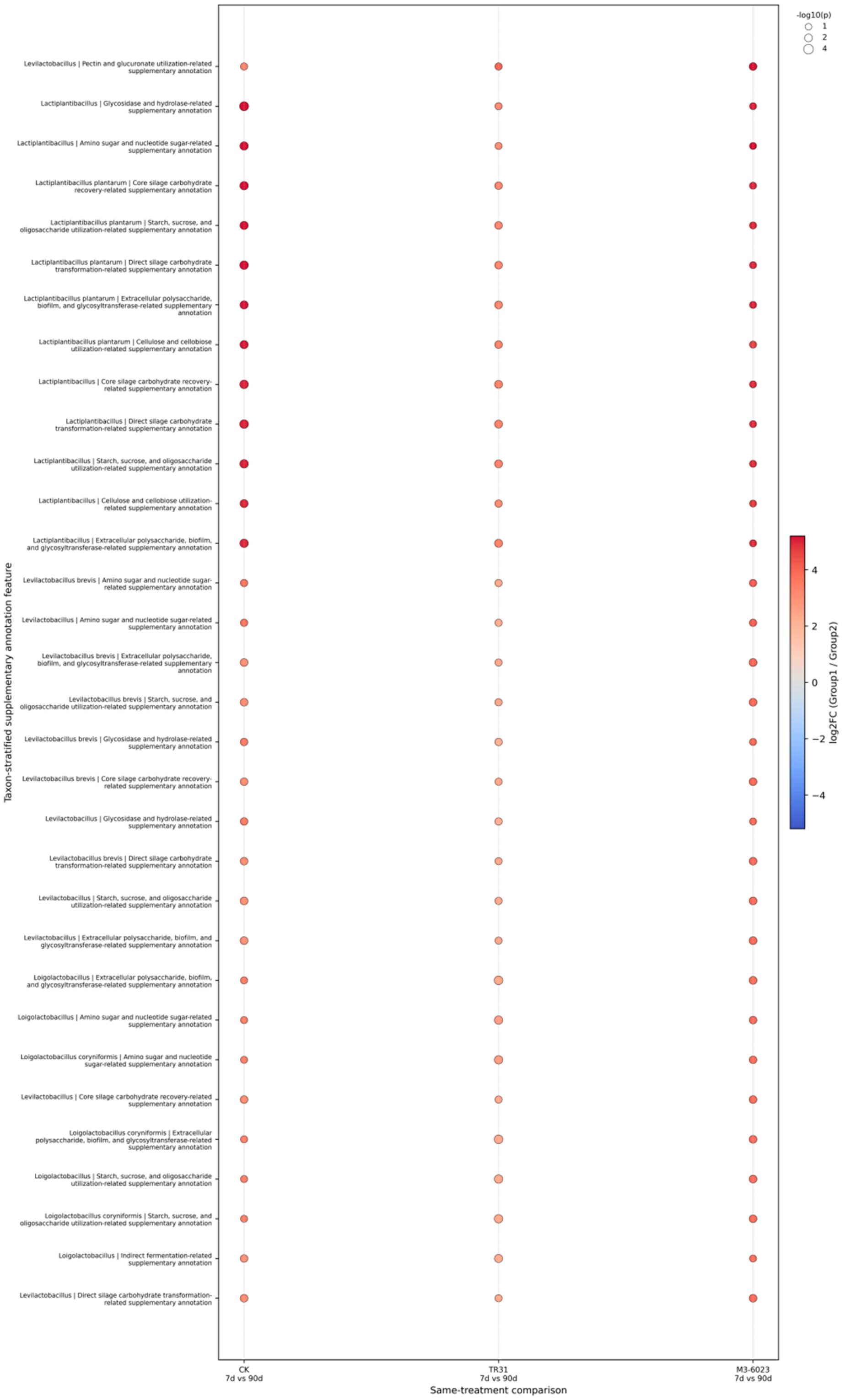

Figure S7. Same-treatment stage-associated patterns in selected taxon-linked CAAC-supplemented functional categories.

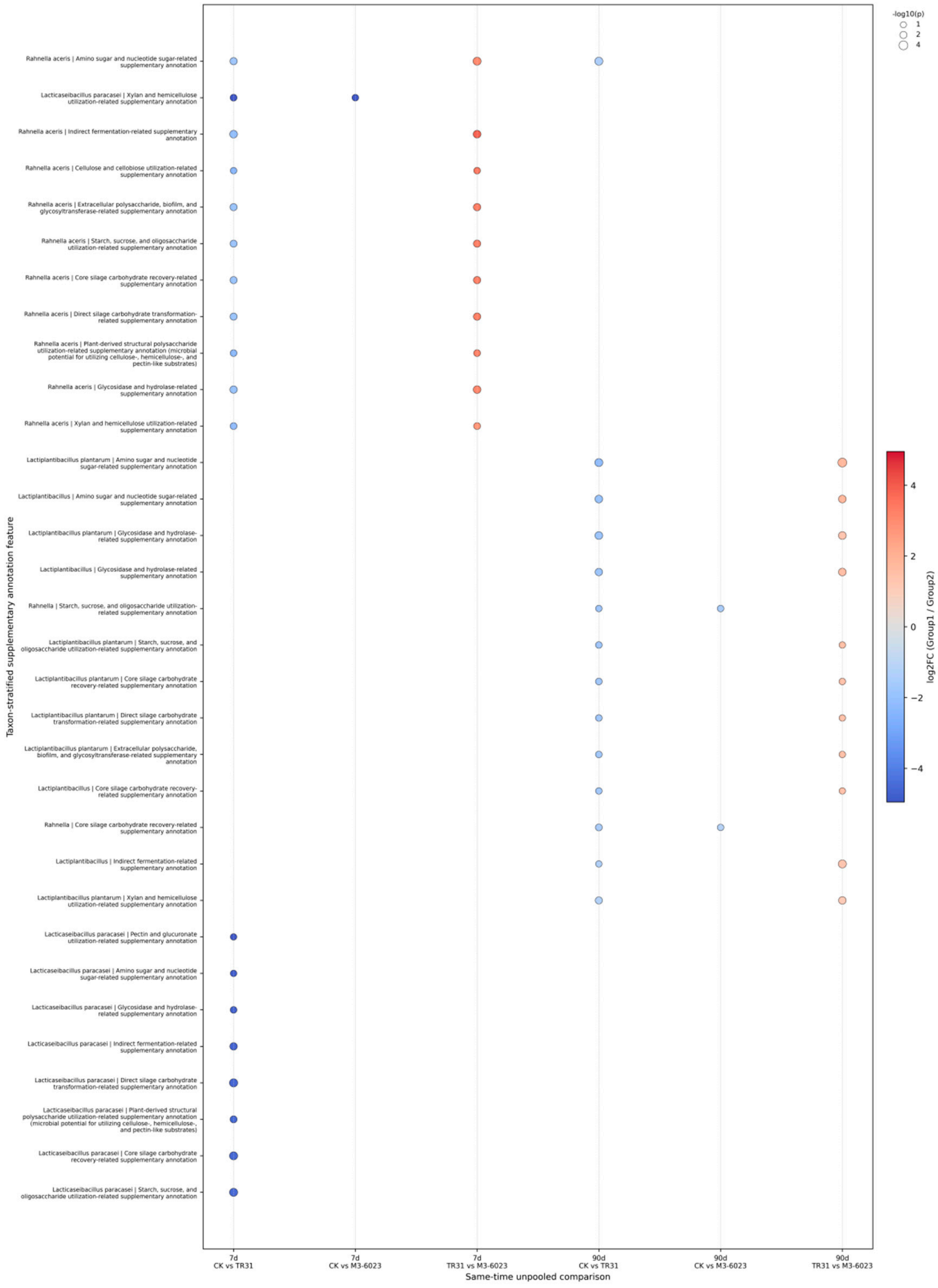

Figure S8. Same-time inoculation-associated patterns in selected taxon-linked CAAC-supplemented functional categories.

Table S1. Sensitivity analyses for confidence-score parameter selection.

(A) Confidence-score weight ablation results. Fifteen weight combinations were evaluated under the constraint Wc+We≤1.0. AP, best F1, and best threshold are reported for each combination.

Wc	We	AP	Best F1	Best Threshold
0.6	0.0	0.9805	0.9835	0.1200
0.6	0.1	0.9859	0.9835	0.2042
0.6	0.2	0.9859	0.9835	0.2883
0.6	0.3	0.9859	0.9835	0.3725
0.6	0.4	0.9859	0.9835	0.4567
0.7	0.0	0.9805	0.9835	0.1400
0.7	0.1	0.9859	0.9835	0.2242
0.7	0.2	0.9859	0.9835	0.3083
0.7	0.3	0.9859	0.9835	0.3925
0.8	0.0	0.9805	0.9835	0.1600
0.8	0.1	0.9859	0.9835	0.2442
0.8	0.2	0.9859	0.9835	0.3283
0.9	0.0	0.9805	0.9835	0.1800
0.9	0.1	0.9859	0.9835	0.2642
1.0	0.0	0.9805	0.9835	0.2000

(B) Sensitivity of confidence-tier assignment to the neighbor-similarity baseline.

Similarity baseline	Tier 1 count	Tier 2 count	Tier 3 count	Tier Low count	All integrable KO–EC features
0.85	303,571	251,905	242,986	0	140
0.90	303,505	251,970	242,986	1	140
0.95	168,454	311,123	117,385	201,500	136

Note: "All integrable KO－EC features" refers to all KO－EC features that satisfied the annotation-validity and EC-to-KO mapping criteria before silage-relevance

filtering. This number differs from the 102 silage-relevant KO－EC features reported in the main text, which were obtained after applying the silage-relevance filtering rules described in Section 2.5.2. The 0.90 baseline was retained because it produced a stable tier distribution similar to 0.85 while avoiding the excessive Tier Low reassignment observed under the stricter 0.95 setting.

Table S2. Actual precision of each tier on the cross-validation test set. Tier-specific precision, recall, F1, and FPR were calibrated on the 5-fold cross-validation test set comprising 291,488 positive predictions with ground-truth labels.

Tier	Threshold Range	Samples	Precision	Recall	F1	FPR
Tier 1	≥0.60	278,702	97.16%	100.00%	0.9856	2.84%
Tier 2	0.40 ≤ score < 0.60	9,521	88.52%	100.00%	0.9391	11.48%
Tier 3	0.25 ≤ score < 0.40	3,265	86.25%	100.00%	0.9262	13.75%
Tier Low	<0.25	0	N/A	N/A	N/A	N/A

Table S3. Curated non-exclusive silage-related functional categories summarized from CAAC-supplemented annotations

Category ID	Display name used in this study	Rule basis for category assignment	No. of KO–EC features	Interpretation in this study
M01	Direct silage carbohydrate-related supplementary annotations	Features classified as Direct_silage_carbohydrate based on silage-relevance annotation	56	CAAC-supplemented annotations directly associated with carbohydrate-related silage functions
M02	Auxiliary fermentation-associated supplementary annotations	Features classified as Indirect_fermentation_related	25	Supplementary annotations indirectly associated with fermentation-relevant carbohydrate or glycan-related processes
M03	Starch-, sucrose-, and	Terms including	26	Supplementary

	oligosaccharide-associated supplementary annotations	starch, sucrose, maltose, dextrin, glycogen, amylase, glucosidase, fructofuranosidase, or cellobiose		annotations associated with soluble or storage carbohydrate-related substrates
M04	Cellulose- and cellobiose-associated supplementary annotations	Terms including cellulose, cellulase, endoglucanase, beta-glucosidase, or cellobiose	10	Supplementary annotations associated with cellulose- or cellobiose-related substrates
M05	Xylan- and hemicellulose-associated supplementary annotations	Terms including xylan, xylosidase, xylanase, arabinose, arabinan, arabinofuranosidase, hemicellulose, or acetylxylan	7	Supplementary annotations associated with xylan- or hemicellulose-related substrates
M06	Pectin- and glucuronate-associated supplementary annotations	Terms including pectin, pectate, pectinesterase, polygalacturonase, or glucuronate	5	Supplementary annotations associated with pectin- or glucuronate-related substrates
M07	Glycoside hydrolase-associated supplementary annotations	Glycosidase or hydrolase-related terms, or EC numbers in the 3.2.1.- series	50	Supplementary annotations associated with glycoside hydrolase-type activities
M08	Amino sugar- and nucleotide sugar-associated supplementary annotations	Terms including amino sugar, nucleotide sugar, N-acetylglucosamine, chitin, peptidoglycan, or related keywords	11	Supplementary annotations associated with amino sugar or nucleotide sugar metabolism
M09	Other putative glycan-associated supplementary annotations	Terms associated with glycosphingolipid, sphingolipid, glycosaminoglycan, or other cautionary	21	Cautionary category for putative glycan-related supplementary annotations; not

		glycan-related annotations		used as a core silage mechanism
M10	Exopolysaccharide-, biofilm-, and glycosyltransferase-associated supplementary annotations	Terms including biofilm, EPS, exopolysaccharide, dextransucrase, levansucrase, glycosyltransferase, or cellulose synthase	35	Supplementary annotations associated with polysaccharide assembly, glycosyl transfer, or biofilm/EPS-related processes
M11	Plant-derived structural polysaccharide substrate-associated supplementary annotations	Union category including features assigned to M04, M05, or M06	22	Summary category for microbial annotations associated with plant-derived structural polysaccharide substrates; not interpreted as plant-origin genes
M12	Core carbohydrate-related CAAC-supplemented annotation set	Union category including features assigned to M03, M04, M05, M06, or M07	66	Summary category for major carbohydrate-related CAAC-supplemented annotations; not interpreted as an independent metabolic pathway

Table S4. Representative KO－EC evidence supporting curated category assignment

Category ID	Display name	Representative KO–EC evidence	Supporting annotation terms or pathway evidence
M01	Direct silage carbohydrate-related supplementary annotations	K00688|2.4.1.1, glycogen phosphorylase; K00695|2.4.1.13, sucrose synthase	Starch and sucrose metabolism; silage-relevance keywords including starch and sucrose
M02	Auxiliary fermentation-associate	K01183|3.2.1.14, chitinase;	Amino sugar and nucleotide sugar

Category ID	Display name	Representative KO–EC evidence	Supporting annotation terms or pathway evidence
	d supplementary annotations	K01443|3.5.1.25, N-acetylglucosamine-6-phosphate deacetylase	metabolism; indirect fermentation-related annotation
M03	Starch-, sucrose-, and oligosaccharide-associated supplementary annotations	K00688|2.4.1.1, glycogen phosphorylase; K01176|3.2.1.1, alpha-amylase	Starch and sucrose metabolism; amylase, glucosidase, sucrose, maltose, and related terms
M04	Cellulose- and cellobiose-associated supplementary annotations	K00694|2.4.1.12, cellulose synthase; K01188|3.2.1.21, beta-glucosidase	Cellulose, cellobiose, beta-glucosidase, endoglucanase, and related terms
M05	Xylan- and hemicellulose-associated supplementary annotations	K05972|3.1.1.72, acetylxylan esterase; K15920|3.2.1.37, xylan 1,4-beta-xylosidase	Xylan, xylosidase, acetylxylan, arabinose, arabinofuranosidase, and hemicellulose-related terms
M06	Pectin- and glucuronate-associated supplementary annotations	K22539|4.2.2.2, pectate lyase; K01051|3.1.1.11, pectinesterase	Pectate, pectin, pectinesterase, polygalacturonase, glucuronate, and pentose/glucuronate-related terms
M07	Glycoside hydrolase-associated supplementary annotations	K01176|3.2.1.1, alpha-amylase; K01188|3.2.1.21, beta-glucosidase	Glycosidase/hydrolase-related descriptions and EC 3.2.1.- enzyme annotations
M08	Amino sugar- and nucleotide sugar-associated supplementary annotations	K01183|3.2.1.14, chitinase; K01443|3.5.1.25, N-acetylglucosamine-6-phosphate deacetylase	Amino sugar and nucleotide sugar metabolism; N-acetylglucosamine, chitin, and

Category ID	Display name	Representative KO–EC evidence	Supporting annotation terms or pathway evidence
			peptidoglycan-related terms
M09	Other putative glycan-associated supplementary annotations	K12373|3.2.1.52, hexosaminidase; K01186|3.2.1.18, sialidase	Other glycan degradation, sphingolipid, glycosaminoglycan, and glycosphingolipid-related annotations
M10	Exopolysaccharide-, biofilm-, and glycosyltransferase-associated supplementary annotations	K00694|2.4.1.12, cellulose synthase; K06119|2.4.1.-, sulfoquinovosyltransferase	Glycosyltransferases, biofilm-related terms, EPS-related terms, cellulose synthase, and polysaccharide-associated transferase annotations
M11	Plant-derived structural polysaccharide substrate-associated supplementary annotations	K00694|2.4.1.12, cellulose synthase; K05972|3.1.1.72, acetylxylan esterase	Union of cellulose/cellobiose-, xylan/hemicellulose-, and pectin/glucuronate-associated categories
M12	Core carbohydrate-related CAAC-supplemented annotation set	K00688|2.4.1.1, glycogen phosphorylase; K01188|3.2.1.21, beta-glucosidase	Union of starch/sucrose/oligosaccharide-, cellulose/cellobiose-, xylan/hemicellulose-, pectin/glucuronate-, and glycoside hydrolase-associated categories

Table S5. Pathway coverage changes before and after backfilling

Metric	Before	After	Change
Mean pathway coverage (%)	14.48	14.80	+0.32

Pathways with gain	—	64/564	11.3%
Pathways unchanged	—	500/564	88.7%
Maximum single-pathway gain (%)	—	25.0	—
Carbohydrate metabolism pathways with gain	—	25/47	53.2%
Newly activated sample – pathway occurrences	—	146	—
Dataset-level newly detected carbohydrate-metabolism pathways	0/47	2/47	4.3%

Table S6. Annotation-derived pathway co-occurrence topology before and after CAAC supplementation

Parameter	Pre-backfilling	Post-backfilling	Change
Nodes	408	413	+5 (+1.2%)
Edges	13,717	12,443	-1,274 (-9.3%)
Network density	0.1652	0.1463	-0.0180 (-11.4%)
Annotation-dependent edge differences	—	1,087	8.7%

Table S7. Group-mean TPM-weighted abundance of Lacticaseibacillus paracasei-linked CAAC-supplemented carbohydrate-related categories

Functional category assigned to Lacticaseibacillus paracasei	CK 7 d	TR-31 7 d	M3-6023 7 d	CK 90 d	TR-31 90 d	M3-6023 90 d
Direct silage carbohydrate-related supplementary annotations	29.97	722.39	2504.56	126.83	1841.51	1599.63
Starch-, sucrose-, and oligosaccharide-associated supplementary annotations	27.95	628.64	2230.48	118.20	1601.44	1347.11
Cellulose- and	7.72	139.45	352.42	12.73	357.07	289.10

cellobiose-associated supplementary annotations						
Xylan- and hemicellulose-associated supplementary annotations	1.70	79.48	200.13	6.27	195.03	185.84
Pectin- and glucuronate-associated supplementary annotations	0.00	3.12	23.73	1.18	5.31	31.86
Glycoside hydrolase-associated supplementary annotations	4.40	114.70	398.33	24.45	259.41	228.33
Plant-derived structural polysaccharide substrate-associated supplementary annotations	9.42	222.05	576.28	20.19	557.41	506.80
Core carbohydrate-related CAAC-supplemented annotation set	31.12	732.96	2582.63	134.86	1851.12	1619.87

Table S8. Fermentation characteristics and chemical composition of CK and Lacticaseibacillus paracasei-inoculated treatment groups after 7 and 90 days of ensiling.

Parameter	Ensiling Day	CK	M3-6023	TR-31	SEM	P-value (Day)	P-value (Treatment)	P-value (T×D)
pH	7	3.97a	4.08b	4.02ab	0.014	<0.001	0.006	<0.001
	90	3.7a	3.69ab	3.72ab				
DM (%)	7	355a	349a	342a	4.93	0.439	0.037	0.845
	90	357a	355a	343a				
LA (g/kg)DM	7	476b	320c	227c	30.96	<0.001	0.014	<0.001
	90	552a	663a	606a				
AA (g/kg)DM	7	149.9bc	133.8bc	55.8c	30.08	<0.001	0.042	0.768
	90	245.8a	258.7a	194.9a				
LA/AcA	7	3.17ab	2.66ab	4.07a	0.342	0.091	0.033	0.261
	90	2.4b	2.83ab	3.17ab				

Ethanol(g/kg)DM	7	56.6b	34.1d	29.1d	3.79	<0.001	<0.001	0.022
	90	63.1a	63.7a	44.4c				
WSC (g/kg)DM	7	155c	185b	192b	3.51	<0.001	<0.001	0.001
	90	257a	273a	263a				
NH_3-N (mmol/L)	7	140b	129b	117b	5.90	<0.001	0.182	0.109
	90	161a	169a	163a				
CP (g/kg)DM	7	54.5c	61.4b	69.5a	1.45	0.210	0.043	<0.001
	90	68.5a	61.4b	60.3b				
NDF (g/kg)DM	7	303c	254c	273c	11.66	0.002	<0.001	<0.001
	90	220d	377a	336b				
ADF (g/kg)DM	7	191b	129c	124c	8.08	<0.001	0.320	<0.001
	90	121c	191b	213a				

Table S9. Representative CAAC-supplemented KO–EC features supporting the exploratory case study.

KO	EC	Function	Category	Original status	CAAC evidence	Clearest pattern
K00688	2.4.1.1	Glycogen phosphorylase	Starch/sucrose	Present	222,836 records; TPM 749,028; 18 samples	M3-6023_7d
K00695	2.4.1.13	Sucrose synthase	Starch/sucrose	Absent in case-study Pre-CAAC table	135,815 records; TPM 429,688; 18 samples	M3-6023_7d
K01176	3.2.1.1	Alpha-amylase	Starch/sucrose	Present	39,419 records; TPM 135,579; 18 samples	M3-6023_7d
K01188	3.2.1.21	Beta-glucosidase	Cellulose/cellobiose	Low/incomplete	7,088 records	Ensiled samples
K01179	3.2.1.4	Endoglucanase	Cellulose/cellobiose	Present	4,195 records; TPM 12,054; 18 samples	CK_7d
K05972	3.1.1.72	Acetylxylan esterase	Xylan/hemicellulose	Low/incomplete	16,123 records	90 d pattern
K15920	3.2.1.37	Xylan 1,4-beta-xylosidase	Xylan/hemicellulose	Low/incomplete	4,769 records	Xylan-related pattern
K01051	3.1.1.11	Pectinesterase	Pectin/glucuronate	Present	TPM 7,772; 18 samples	CK_7d

K22539	4.2.2.2	Pectate lyase	Pectin/glucuronate	Low/incomplete	3,161 records	Pectin-related pattern
